# Deciphering the Therapeutic Effect of Quercetin-Loaded Pumpkin Seed Oil Nanoemulsion on Rheumatoid Arthritis in a Rat Model

**DOI:** 10.3390/biomedicines14102253

**Published:** 2026-10-05

**Authors:** Hanan Hendawy, Mohamed Korish, Nourhan Gamal El-Rahmany, Ibrahim Elsayed Helal, W. El Hotaby, Fatma M. Khodary, Safaa M. Awad

**Affiliations:** 1 Department of Veterinary Surgery, Faculty of Veterinary Medicine, Suez Canal University, Ismailia 41522, Egypt; 2 Department of Agriculture, Faculty of Environment Sciences, King Abdulaziz University, Jeddah 80269, Saudi Arabia; mkorish@kau.edu.sa (M.K.); ihelal@kau.edu.sa (I.E.H.); 3 Biochemistry and Nutrition Department, Faculty of Women for Arts, Science and Education, Ain Shams University, Cairo 11757, Egypt; safaa.mostafa@women.asu.edu.eg; 4 Spectroscopy Department, National Research Center, 33 El-Bohouth St., Dokki, Giza 12622, Egypt; walid.mosad@gmail.com; 5 Department of Cytology and Histology, Faculty of Veterinary Medicine, Suez Canal University, Ismailia 41522, Egypt; vet.fatmaa@vet.suez.edu.eg

**Keywords:** PSONE, OPG/RANKL, miRNA-146a, RA, Pyroptosis, JAK/STAT, NFκβ

## Abstract

**Background:** Rheumatoid arthritis (RA) is a chronic systemic autoimmune disorder characterized by persistent synovitis, progressive joint destruction, and significant morbidity. Despite advances in targeted therapies, achieving sustained remission remains challenging, necessitating the exploration of novel, multi-targeted therapeutic strategies. This study investigates the therapeutic potential of a self-emulsifying nanoformulation, quercetin-loaded pumpkin seed oil nanoemulsion (Qu-PSONE), in a complete Freund’s adjuvant (CFA)-induced RA rat model, focusing on elucidating its integrated molecular mechanisms. **Methods:** Adult male Wistar rats (n = 50) were divided into a normal control group (n = 10) and an RA-induced rat model (n = 40), with the latter being further subdivided into four experimental groups: RA control, free quercetin (Qu), pumpkin seed oil nanoemulsion (PSONE), and Qu-PSONE-treated groups. We performed comprehensive analyses, including physicochemical characterization of the nanoformulations and quantification of synovial protein levels of key mediators (IL-17, TLR-4, NF-κBp65, Nrf2, OPG, RANKL, JAK1, and STAT3) via ELISA. In parallel, synovial gene expression of *GSDMD* and miRNA-146a was assessed using RT-qPCR. Treatment efficacy was further evaluated through paw thickness measurements, clinical arthritis scoring, radiographic assessment of joint erosion, and histopathological examination, alongside immunohistochemical staining for TNF-α and MMP-9. **Results:** Our findings demonstrate that Qu-PSONE exerts a superior nutrigenomic inhibitory effect on the inflammatory cascade and joint deformity associated with CFA-induced adjuvant arthritis. The Qu-PSONE significantly modulated miRNA-146a expression, serving as a sensitive indicator of attenuated synoviocyte activation. This treatment concurrently increased Nrf2 synovial level, suppressed synovial NF-κBp65, TLR-4, and IL-17 levels, which in turn downstream-inhibited the JAK/STAT pathway, balanced RANKL/OPG signaling, and downregulated transcriptional priming of the pyroptotic marker *GSDMD*. Together, these findings demonstrate that Qu-PSONE effectively breaks the inflammatory cascade in adjuvant arthritis. These palliative effects of Qu-PSONE were robustly correlated with reduced paw edema, improved arthritis scores, mitigated radiographic joint damage, and diminished synovial infiltration and MMP-9 expression, as confirmed by histological and immunohistochemical analyses. **Conclusions:** Qu-PSONE halts arthritic progression and curtails joint damage through multitarget suppression of the NF-κBp65/TLR-4/IL-17 inflammatory axis at the synovial level. This response is accompanied by reduced JAK1 and STAT3 protein expression, re-established RANKL/OPG homeostasis to mitigate bone destruction, and attenuated *GSDMD* transcript levels, with miRNA-146a serving as a responsive biomarker of disease resolution.

## 1. Introduction

Rheumatoid arthritis (RA) is a chronic autoimmune inflammatory disease that causes symmetrical polyarthritis with joint swelling, pain, and stiffness [1]. RA is characterized by synovial membrane invasion and joint damage. Pathological studies show involvement of both joint and extra-articular organs, including the eye, skin, nervous system, heart, kidney, lung, and digestive system [2]. Patients with recurrent rheumatoid arthritis exhibit general symptoms such as loss of appetite, anemia, and discomfort, which have an impact on their quality of life. The cause of rheumatoid arthritis is unknown; however, environmental and genetic factors are established risk factors for disease development [3]. Cytokine network imbalance plays a significant role in RA pathogenesis. An imbalance between proinflammatory and anti-inflammatory cytokines induces autoimmunity, persistent inflammation, and thus joint degeneration. Regulating TNF-α and IL-1β, two important proinflammatory cytokines, is crucial in RA [4]. Understanding the role of cytokines in arthritis and osteoclastogenesis, including IL-17, IL-18, and the receptor activator of nuclear factor κB (RANKL) ligand, can help improve treatment options. Furthermore, cytokines are considered potentially important triggers of RA; thus, targeting cytokine balance in RA may improve both diagnosis and treatment [5].

Adjuvant-induced arthritis (AIA) in rats is a chronic inflammatory condition characterized by synovial membrane invasion and joint damage. Clinically, pathologically, immunologically, and histologically, it more closely resembles human RA. AIA not only contributes to our understanding of RA pathogenesis in humans, but it also helps to create new RA therapies [6]. This study used complete Freund’s adjuvant (CFA)-induced arthritis as an animal model to evaluate the ameliorative effects of the various tested medications. Alternative, safer, and more effective natural product-based treatments are necessary because of the adverse effects and toxicity of frequently prescribed anti-arthritic pharmaceuticals [7].

Quercetin, a flavonoid present in fruits and vegetables, is known for its anti-inflammatory and antioxidant properties. Quercetin has potential therapeutic effects due to its anti-inflammatory and antioxidant activities; it mitigates several inflammatory diseases. Quercetin can be found in grapes, onions, berries, apples, and tomatoes [8].

One of the primary goals of nanomedicine is to improve the therapeutic effect of active pharmacological medicines for treating chronic diseases. Pumpkin seeds are rich in bioactive elements such as β-carotene, unsaturated fatty acids, phenolic compounds, phytosterols, and tocopherols, particularly in the oil compartment. These functional dietary elements have been shown to have both anti-inflammatory and antioxidant properties [9]. In recent years, nanoemulsion drug delivery systems, an emulsification technology, have gained attention because they can improve the oral bioavailability of hydrophobic medicines and bioactive food components and support a variety of drug-delivery applications [10].

Nanoemulsions are better than microemulsions due to their smaller size, increased surface area, and lack of typical creaming, flocculation, coalescence, or precipitation. Nanoemulsions are isotropic mixtures of oil, surfactants, and co-surfactant components that spontaneously form emulsions when exposed to gastrointestinal fluid [11]. In addition to improving stability, these lipid-based nanoparticles, when used as novel drug delivery systems (DDSs), have been shown to improve drug solubility and dissolution in the gastrointestinal tract (GIT), increasing absorption of poorly water-soluble drugs [12].

Gasdermin D (GSDMD), a key executor of pyroptosis and inflammatory cell death, has been implicated in the pathogenesis of rheumatoid arthritis (RA). Upon proteolytic activation, GSDMD forms plasma membrane pores that release proinflammatory cytokines, contributing to joint tissue damage and synovial inflammation [13]. The interplay between inflammation and osteoclastogenesis plays a central role in rheumatoid arthritis pathogenesis. While microRNA-146a acts as a negative-feedback regulator to restrain NF-κB signaling, it is chronically upregulated during active arthritis as a compensatory stress response. Consequently, its expression serves as a sensitive molecular indicator of ongoing inflammatory burden [14]. Thus, this study investigates the relationship between miRNA-146a, NF-κBp65, and increased inflammatory cytokines. Here, we examine the effects of natural treatments that may counter inflammatory signaling and RA pathogenesis.

This study evaluates the anti-inflammatory, antioxidant, and anti-arthritis potential of free quercetin, pumpkin seed oil nanoformulation, and a quercetin-loaded pumpkin seed oil nanoformulation in a CFA-induced rheumatoid arthritis model, as well as their regulatory effects on pyroptosis-associated markers such as GSDMD. To our knowledge, this is the first study to investigate whether a quercetin-loaded pumpkin seed oil nanoformulation can attenuate CFA-induced adjuvant arthritis through the multitarget regulation of miRNA-146a, GSDMD transcriptional pathways, and RANKL/OPG bone homeostasis.

## 2. Materials and Methods

### 2.1. Chemicals

Pumpkin seed oil was purchased from the local market (IMTENAN, Cairo, Egypt). Complete Freund’s adjuvant (CFA), Quercetin, Tween 80, ethanol, saline, and polyethylene glycol (PEG 400) were obtained from Sigma-Aldrich Co., St. Louis, MO, USA.

### 2.2. Preparation of Pumpkin Seed Oil and Quercetin-Loaded Pumpkin Seed Oil Nanoformulation

The lipid-based nanoformulation was prepared as a Self-Nanoemulsifying Drug Delivery System (SNEDDS) according to previously reported protocols by Jafari et al. [8] and Yazdi et al. [9], with slight modifications. Briefly, the anhydrous preconcentrate was formulated by mixing 30% *v*/*v* Pumpkin Seed Oil (PSO), 50% *v*/*v* Tween 80, and 20% *v*/*v* PEG 400 under continuous stirring at 25 °C until a homogeneous mixture was obtained. For the drug-loaded formulation. Quercetin was dissolved in the vehicle at a final concentration of 10 mg/mL (1 g in 100 mL). The resulting clear mixture is an anhydrous self-nanoemulsifying preconcentrate that spontaneously reconstitutes into a fine oil-in-water (*o*/*w*) nanoemulsion upon dispersion in aqueous fluids (deionized water for in vitro characterization or gastrointestinal fluids for in vivo oral administration). After cooling to room temperature, the mixture was sonicated with a probe sonicator for 10 min, yielding a self-nanoemulsified drug delivery system, which was then stored at 4 °C. The corresponding quercetin-free (blank) nanoemulsion was prepared following the identical protocol without active drug loading. The Nanomaterials Research and Synthesis Unit (Dokki, Giza, Egypt) conducted the formulation process.

### 2.3. Physicochemical and Morphological Characterization of Nanoformulation 

Dynamic light scattering and zeta potential measurements: The average hydrodynamic diameter, polydispersity index (PDI), and zeta potential of the nanoformulation were assessed using a Microtrac Wave particle size and zeta potential analyzer (software version 12.0.1.0). Conductivity measurements were simultaneously recorded. All measurements were performed at 25 °C after appropriate dilution with deionized water to avoid multiple scattering effects.

Transmission electron microscopy: The morphology of the nanoemulsion droplets was visualized using HR-TEM (JEOL JEM-2100, Tokyo, Japan). Before imaging, the sample was mildly sonicated for 5 min in an ultrasonic bath (Crest Ultrasonics Corp., NJ, USA) to ensure uniform dispersion. A small aliquot was then deposited onto a carbon-coated copper grid and allowed to dry at ambient temperature. To enhance image contrast, the dried grid was negatively stained with a 1% (*w*/*v*) phosphotungstic acid solution for a few minutes; excess stain was removed, and the grid was air-dried before examination [9].

### 2.4. Experimental Design

Fifty healthy adult male Wistar rats, aged 10–12 weeks and weighing 220–250 g, were obtained from the Laboratory Animal House, Faculty of Veterinary Medicine, Suez Canal University, Egypt, and used in the present study. Rats underwent a 1-week adaptation period and were kept in standard polypropylene cages at 22 ± 2 °C and 55 ± 5% humidity, with a 12/12-h light/dark cycle. Rats were fed a basal diet [15] and water, which were supplied ad libitum. This study is reported in accordance with the ARRIVE 2.0 guidelines for reporting animal research. The Scientific Research Ethics Committee for the Care and Use of Experimental Animals in Education and Scientific Research (SCU-VET-AREC), Faculty of Veterinary Medicine, Suez Canal University, Egypt, approved this study under code number (approval no. SCU-VET-AREC-R-2025035) dated 11 September 2025. To minimize potential sources of bias and confounding, rats were maintained under identical housing and husbandry conditions throughout the study, including standardized temperature, relative humidity, light/dark cycle, diet, and access to water. Rats were housed under comparable cage conditions, and experimental procedures and outcome assessments followed a standardized protocol with consistent timing across all groups. Rats were eligible for enrollment if they were healthy adult male Wistar rats aged 10–12 weeks, weighing 220–250 g, showed no clinically apparent abnormalities, and completed the 1-week acclimatization period. Arthritis-confirmation criteria were applied only to CFA-injected animals. No animals were excluded or died prematurely, and all 50 rats completed the experimental period.

We randomly allocated rats to five experimental groups (n = 10/group) using a computer-generated randomization sequence before confirming arthritis. Because randomization occurred before CFA administration to ensure allocation concealment, arthritis confirmation was not a prerequisite for randomization; however, all CFA-injected animals developed the arthritic phenotype, and no animals were excluded.

Sample size was established a priori at a total of 50 animals (10 animals per group across 5 experimental groups). To confirm the adequacy of this sample size for the planned linear mixed-effects models, a sensitivity power analysis was conducted using G*Power (version 3.1.9.7) based on a repeated-measures design. This analysis verified that a total sample size of 50 provides 80% statistical power (a = 0.05) to detect effect sizes f = 0.22, ensuring robust and sensitive detection of changes in primary arthritic endpoints over time. Allocation concealment was maintained through an independently held allocation list, which remained inaccessible to the investigators responsible for assigning animals until each assignment was made. Personnel administering treatments and investigators performing clinical, radiographic, histopathological, and immunohistochemical assessments were unaware of group allocation during their respective activities. Personnel conducting statistical analyses were also blinded to group identity. Measurements were performed in the same sequence and under comparable environmental conditions whenever possible.

### 2.5. Induction of CFA-Induced Adjuvant Arthritis 

Arthritis was produced by administering a single dose of CFA to the left hind paw [16]. Before CFA injection, rats were anesthetized with ketamine (80 mg/kg) and xylazine (10 mg/kg), administered intraperitoneally. The plantar surface was disinfected with 70% ethanol and straightened. CFA (0.1 mL; 10 mg/mL heat-killed Mycobacterium tuberculosis) was mixed thoroughly before use and drawn into a 1-mL insulin syringe fitted with a 27-gauge needle.

The needle was inserted subcutaneously at a shallow angle into the central plantar region, and the CFA was injected slowly to ensure proper deposition. The control group received sterile saline (0.1 mL) using the same procedure. Rats were observed daily for paw swelling and general health. After CFA induction, rats were monitored for clinical signs of arthritis for seven days. CFA-induced Adjuvant Arthritis was confirmed clinically by the presence of paw and ankle joint swelling and erythema, accompanied by increased paw/ankle thickness relative to normal control animals. Arthritis severity was assessed using a predefined clinical arthritis scoring system.

### 2.6. Treatment Protocol

Treatment was initiated on day 7 after CFA induction, following clinical confirmation of arthritis. Treatments and control preparations were administered once daily by oral gavage (1 mL per rat) for 21 days. Baseline paw measurements and arthritis scores were recorded before CFA injection, and follow-up assessments were performed on days 7, 14, and 21 after treatment initiation. Rats had previously been randomly allocated to five experimental groups (n = 10/group), as described in Section 2.4:

- Normal control (NC): Healthy rats received 1 mL of corn oil.
- Arthritis control (RA): CFA-induced arthritic rats received 1 mL of saline to undergo the same daily gavage handling as the treated animals.
- Quercetin-treated arthritis group (RA + Qu): CFA-induced arthritic rats received quercetin dissolved in corn oil at 50 mg/kg body weight in a total volume of 1 mL.
- PSONE-treated arthritis group (RA + PSONE): CFA-induced arthritic rats received a fixed volume of 1 mL of pumpkin seed oil nanoemulsion.
- Qu-PSONE-treated arthritis group (RA + Qu-PSONE): CFA-induced arthritic rats received a fixed volume of 1 mL of quercetin-loaded pumpkin seed oil nanoemulsion.

#### 2.6.1. Animal Care and Monitoring

Buprenorphine (0.05 mg/kg, subcutaneously) was available as rescue analgesia, administered based on a daily composite pain assessment (body weight, grooming, mobility, response to handling). No animal reached the predefined threshold for intervention; thus, no buprenorphine was administered. All rats were handled gently, and injections utilized fine-gauge needles. Rats were monitored daily during acclimatization and at least once daily during induction and treatment, with twice-daily monitoring during the first 72 h post-CFA. Assessments included body weight, behavior, posture, grooming, mobility, food and water intake, signs of pain, and injection site/paw condition (specifically swelling, erythema, joint deformity, and weight bearing). Arthritis severity was scored at scheduled time points. Prespecified humane endpoints included > 20% body-weight loss, inability to access food or water, anorexia > 24 h, severe lethargy/recumbency, uncontrolled pain, self-mutilation, or persistent hunched posture with piloerection > 24 h; rats approaching these endpoints were monitored twice daily. Ultimately, no unexpected adverse events, premature deaths, animal exclusions, or humane endpoints occurred.

#### 2.6.2. Euthanasia and Tissue Collection

On day 21 after treatment initiation, following radiographic examination, rats were euthanized by inhalational overdose of isoflurane (5% in oxygen) delivered through a calibrated vaporizer into an induction chamber. Deep anesthesia was confirmed by loss of the righting reflex, absence of a response to a firm pedal stimulus, and absence of spontaneous movement. Isoflurane exposure was continued until respiratory arrest occurred. Death was confirmed by the absence of spontaneous respiration, heartbeat, and response to noxious stimulation before removal from the chamber and tissue collection.

Following confirmation of death, the left hind paw joint was dissected, and separate tissue portions were allocated to histopathological, immunohistochemical, and molecular analyses. Before fixation, synovial tissue intended for ELISA and RT-qPCR was microdissected from the same joint, weighed, snap-frozen in liquid nitrogen, and stored at −80 °C. The remaining joint specimen was fixed in 10% neutral buffered formalin for 48 h, decalcified in 10% EDTA, and processed for paraffin embedding and subsequent histopathological and immunohistochemical examination.

For protein analysis, frozen synovial tissue was homogenized in ice-cold phosphate-buffered saline (PBS; pH 7.4; 1:10 *w*/*v*) containing protease and phosphatase inhibitors. Homogenates were centrifuged at 10,000× *g* for 15 min at 4 °C, and the supernatants were collected. Total protein concentrations were determined using a bicinchoninic acid (BCA) assay to normalize subsequent ELISA measurements.

### 2.7. Clinical Evaluation of Paw Swelling 

Paw thickness was measured using a Vernier caliper as an indicator of edema. Baseline measurements were obtained before CFA induction, and subsequent measurements were recorded on days 7, 14, and 21 after treatment initiation. The arthritis score was calculated by the method of [17]. Swelling and erythema of the paws were graded using a 5-point scale: 0, no sign of swelling or erythema; 1, signs of swelling or erythema in the ankle or wrist; 2, signs of swelling or erythema in the ankle and tarsal or wrist and carpal; 3, signs of swelling or erythema extending to the metatarsals or metacarpals; and 4, signs of swelling or erythema involving the entire hind or fore paw.

### 2.8. X-Ray Analysis

At the end of the experimental period (day 21 after treatment initiation), radiographic examinations of the affected hind limbs were performed in all rats to assess structural joint damage associated with adjuvant-induced arthritis. Before radiographic examination, rats were anesthetized with ketamine (80 mg/kg) and xylazine (10 mg/kg) administered intraperitoneally. Digital radiographs were acquired using a high-resolution X-ray imaging system with a film-to-focus distance of 60 inches under standardized exposure conditions (43 kVp, 2 mA). We obtained all images using identical positioning and exposure parameters to ensure consistency across experimental groups. Radiographic images of the adjuvant-injected hind limb were independently evaluated for characteristic pathological features of rheumatoid arthritis, cortical bone erosions, joint space narrowing, joint deformity, and ankylosis, as previously described [18,19]. Radiographic images were evaluated by an investigator blinded to group allocation. Bone erosions were defined as focal areas of cortical bone loss at the articular margins, while joint space narrowing was assessed as a reduction in the distance between adjacent articulating surfaces. Joint deformity was characterized by anatomical misalignment and/or osseous ankylosis.

Radiographic severity was quantified using a semi-quantitative scoring system ranging from 0 to 4 for each parameter, where 0 = normal, 1 = minimal, 2 = moderate, 3 = marked, and 4 = severe pathological changes. The cumulative radiographic score was used to compare the extent of joint destruction among the experimental groups.

### 2.9. Biochemical and Molecular Analysis

#### 2.9.1. Assessment of Synovial TLR4, IL-17, and NF-κB p65 

Total protein levels of Toll-like receptor-4 (TLR4) (Cat# ELK 2106), Interleukin-17 (IL-17) (Cat# ELK 2558), and nuclear factor kappa B p65 (NF-κBp65) (Cat# ELK 1693) were assayed in synovial tissue samples using an ELISA kit (ELK Biotechnology, USA) according to the manufacturer’s instructions.

#### 2.9.2. Estimation of Synovial Nrf2, JAK1, and STAT3 Levels

Total Protein levels of Nuclear factor erythroid 2-related factor 2 (Nrf2) (Nrf2) (Cat# ELK 8043), Janus Kinase-1 (JAK1) (Cat# ELK 7042), and signal transducer and activator of transcription 3 (STAT3) (Cat# ELK9413) were assayed in synovial tissue samples using an ELISA kit (ELK Biotechnology, Sugar Land, TX, USA) according to the manufacturer’s instructions.

#### 2.9.3. Assay of Synovial OPG and RANKL Protein Levels

Osteoprotegerin (OPG) (Cat# ELK10438) and Receptor activator of nuclear factor-κB ligand (RANKL) (Cat# ELK2647) protein levels were assayed in synovial tissue samples using an ELISA kit (ELK Biotechnology, Sugar Land, TX, USA) according to the manufacturer’s instructions.

#### 2.9.4. Quantitative Assessment of Gasdermin D and miRNA-146a Gene Expression

Synovial tissue total RNA was extracted using the RNeasy^®^ Mini Kit (Qiagen, Hilden, Germany) and quantified on a DeNovix spectrophotometer (Wilmington, DE, USA) (A260/A280 ratio = 1.59–1.74). For mRNA quantification, 1 µg of RNA was reverse-transcribed using the FIREScript^®^ RT cDNA Synthesis Kit (Solis BioDyne, Tartu, Estonia), and real-time PCR was executed on a Bio-Rad CFX96 Deep Well™ System with SYBR^®^ Green master mix (Bio-Rad Laboratries, Hercules, CA, USA)across 40 cycles (95 °C activation for 3 min; cycling: 95 °C for 30 s, 59 °C for 40 s, 72 °C for 45 s). Target GSDMD mRNA levels were normalized to GAPDH, whose stability across experimental groups was verified (Table 1). Mature miR-146a-5p expression was evaluated via a stem-loop reverse transcription approach using specific primer sequences (Table 1) normalized to U6 snRNA. All reactions were run in triplicate across n = 10 biological samples per group, alongside non-template controls. Relative fold changes were calculated using the 2−ΔΔCt method based on statistical analyses of raw Δ Ct values using CFX Maestro Software (Bio-Rad Laboratories, Hercules, CA, USA).

### 2.10. Histopathological Analysis (H&E) Staining

The portion of the left hind paw joint retained for histopathological examination after synovial tissue sampling was processed as described in Section 2.6.2. Paraffin-embedded specimens were sectioned at 5 μm and stained with hematoxylin and eosin (H&E) [20].

CFA-induced Adjuvant Arthritis in H&E-stained sections was scored as follows: 0, no pathology; 1, mild articular cartilage degeneration with no inflammatory cells; 2, moderate articular cartilage degeneration with synovial membrane hyperplasia, bone erosion, and a moderate number of inflammatory cells; 3, severe articular cartilage degeneration with bone erosion and a large number of inflammatory cells. We evaluated histopathological changes semiquantitatively in three randomly selected microscopic fields per animal (10 animals per group). We averaged field-level scores to obtain one value per animal for statistical analysis.

### 2.11. Immunohistochemical Analysis

Immunohistochemical analysis was performed to demonstrate TNF-α and MMP-9 [2]. Paraffin-embedded tissue sections of 3 µm thickness were deparaffinized first in xylene and then rehydrated in graded ethanol solutions. The slides were then blocked with 1% bovine serum albumin in Tris-buffered saline or phosphate-buffered saline (pH 7.4) for 2 h, as appropriate. The sections were then immunostained with primary antibodies: rabbit polyclonal anti-TNF-α (ABclonal, Cat # A11534, MA, USA) at a dilution of 1:200 in PBS. For MMP-9 immunostaining, rabbit anti-MMP9 polyclonal antibody (ABclonal, Cat # A11147, MA, USA) was used at a dilution of 1:200 in PBS. After 1 h of incubation, the slides were washed three times in PBS containing 0.5% Triton X-100 for 5 min each, then incubated with anti-rabbit HRP (Thermo Fisher Scientific, Cat # 31460, UK). The sections were then washed again and incubated with 3,3′-diaminobenzidine peroxidase enzyme substrate (DAB) for 30 min as a chromogen and counterstained with Mayer’s hematoxylin [21]. Sections were examined under a light microscope, and the percentage of immunopositive area was quantified using ImageJ software (version 1.33–1.34; National Institutes of Health, Bethesda, MD, USA). Three randomly selected microscopic fields were evaluated per animal (10 animals per group), and field-level measurements were averaged to obtain one value per animal for statistical analysis.

### 2.12. Statistical Analysis

Continuous data are presented as mean ± standard deviation (SD), whereas ordinal and non-normally distributed data are presented as median with interquartile range (IQR). Normality of residuals was assessed using the Shapiro–Wilk test and visual inspection of Q-Q plots. Homogeneity of variance was assessed using Levene’s test. Data that violated these assumptions were analyzed using non-parametric tests (Kruskal–Wallis with Dunn’s post hoc test), as indicated in the figure legends. The individual animal was the experimental unit (n = 10 per group). For histopathological and immunohistochemical assessments, we averaged measurements from three randomly selected microscopic fields per animal to obtain one value per animal before statistical analysis. Technical replicates (performed in triplicate for RT-qPCR) were averaged within each biological sample and were not treated as independent observations.

Paw thickness and arthritis index measurements obtained on days 7, 14, and 21 after treatment initiation were analyzed using linear mixed-effects models, with treatment group, time, and their interaction (group × time) as fixed effects and animal identity as a random intercept to account for within-animal correlation. We included baseline measurements as covariates and adjusted post hoc pairwise comparisons using the Bonferroni method. No data points were missing; therefore, no imputation was required.

We analyzed day-21 post-treatment radiographic scores (bone erosion, joint space narrowing, and joint deformity) and histopathological scores using the Kruskal–Wallis test followed by Dunn’s multiple-comparison test, which controls for multiple pairwise comparisons. Immunohistochemical percentage-positive area was also analyzed using the Kruskal–Wallis test followed by Dunn’s test. Synovial protein levels (IL-17, TLR4, NF-κBp65, Nrf2, OPG, RANKL, JAK1, and STAT3) and relative gene transcript expression (GSDMD and miRNA-146a) were evaluated using one-way ANOVA followed by Tukey’s post hoc test with GraphPad Prism 9.0 (GraphPad Software, NC, USA). Because we analyzed multiple biomarkers, we did not apply a family-wise correction across biomarkers; we analyzed each biomarker as a separate hypothesis. However, within each ANOVA, we used Tukey’s test to control for multiple comparisons among the five groups. For GSDMD and miRNA-146a expression, statistical analyses were performed on ΔCt values, whereas relative expression was displayed as fold changes calculated using the 2−ΔΔCt method. Statistical significance was set at *p* < 0.05 after adjustment for multiple comparisons within each test (Tukey’s test for ANOVA; Dunn’s test for Kruskal–Wallis). No family-wise correction was applied across the different biomarkers.

## 3. Results

### 3.1. Characterization of Nanoformulations

Zeta potential was measured to assess the effect of quercetin on the surface charge of the nanoemulsion dispersed in distilled water. The zeta potential as one of the physicochemical parameters that may contribute to colloidal stability, while recognizing that experimental stability studies are required to substantiate any claim of improved formulation stability. It is worth noting that the surfaces of all samples exhibit zeta potential values, as shown in Table 2. These values play an important role in the stability of the prepared nanoemulsion, where the greatest value indicates a more stable and less aggregated system. Also, a greater magnitude of zeta potential indicates a strong repulsive force between particles that overcomes the attractive forces. As shown in Table 2, the addition of quercetin enhances the Zeta potential from 23.4 to 35 mv.

As evident from Table 2, the prepared nanoemulsion in the presence of quercetin material shows a larger mean particle size (101.6 nm) compared to the sample prepared in the absence of quercetin material (88 nm).

**Figure 1:**
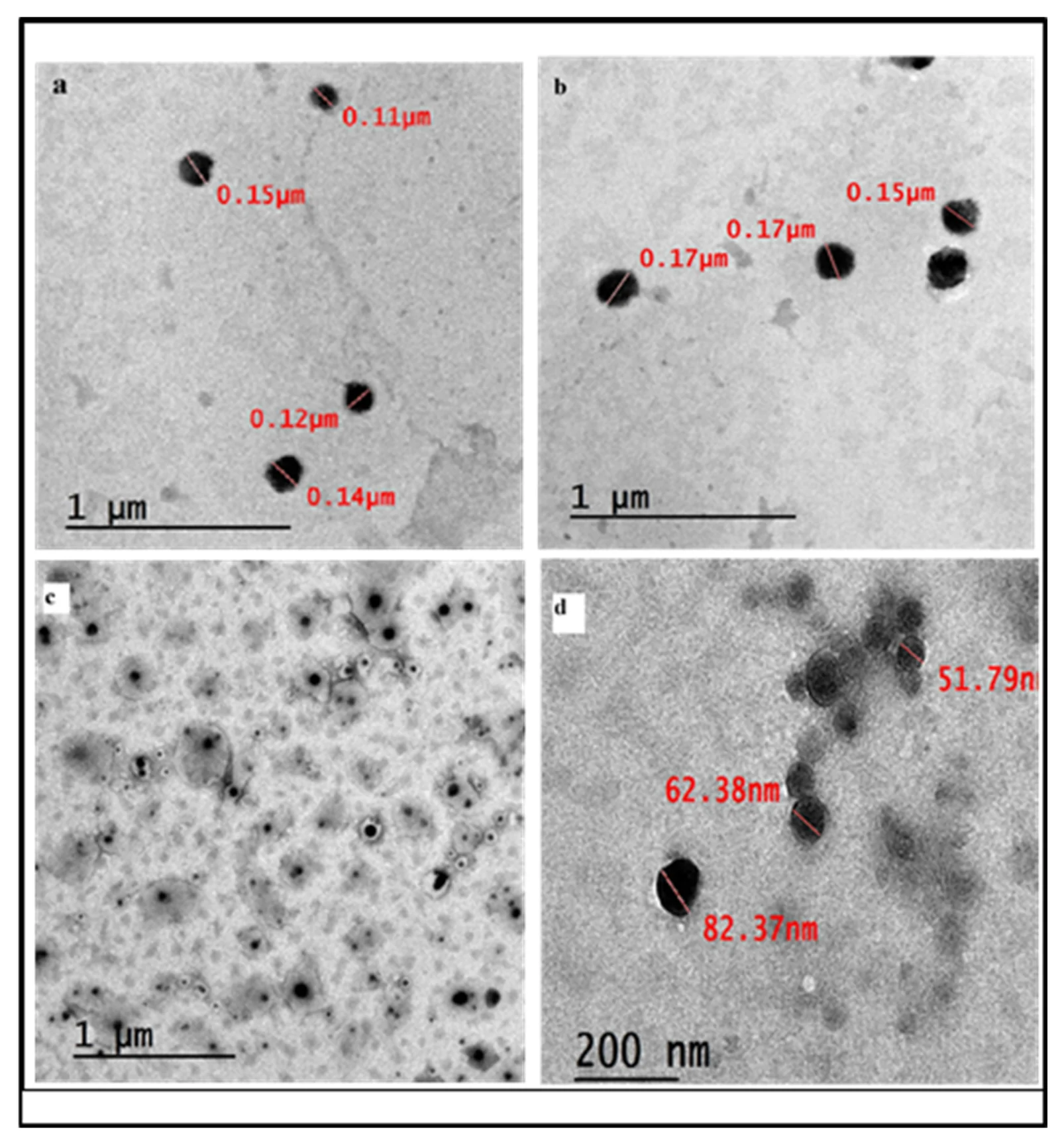
TEM Characterization of Nanoformulations, (**a**,**b**) shows the nanoemulsion particles prepared in the presence of quercetin material. (**c**,**d**) show the nanoemulsion prepared in the absence of quercetin material.

TEM micrographs shown in Figure 1a,b shows the nanoemulsion particles prepared in the presence of quercetin material, which appear almost spherical with a particle size ranging from 110 to 170 nm. These results are similar to those obtained from DLS measurements. Figure 1c,d show the nanoemulsion prepared in the absence of quercetin material; the particles are spherical, and their size ranges from 50 to 90 nm, also these results are complementary in comparison with DLS data. In conclusion, adding quercetin material during nanoemulsion preparation increases particle size and zeta potential, making the particles more stable and decrease its aggregation probability.

### 3.2. Clinical Evaluation of Rat Paw

**Figure 2:**
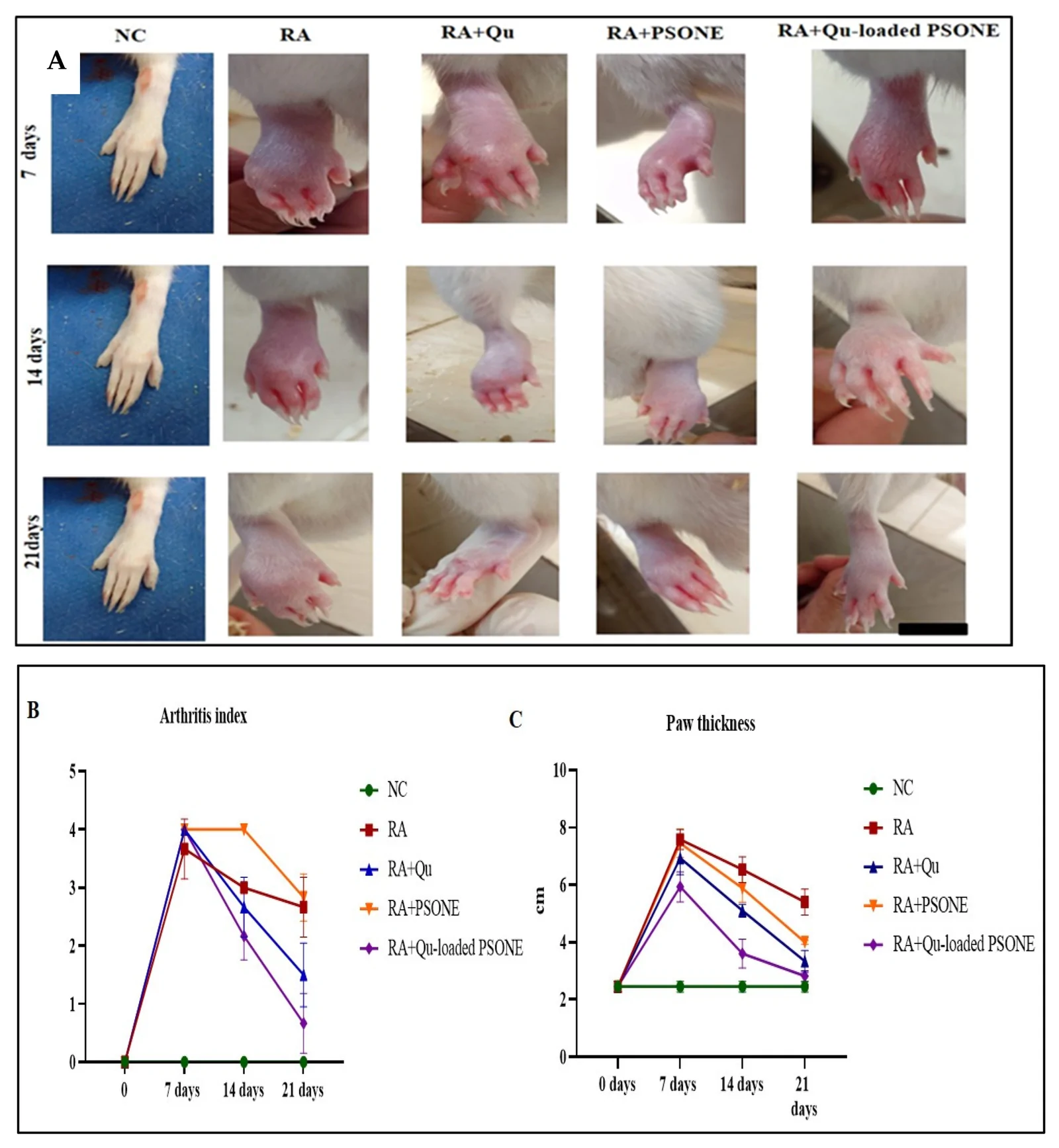
Clinical evaluation of paw inflammation over time. (**A**) Representative gross photographs of rat paws on days 7, 14, and 21 after treatment initiation. The CFA-induced arthritis control group exhibited persistent swelling and erythema, whereas treated groups showed varying degrees of improvement, with the most apparent reduction in visible inflammatory signs in the Qu-PSONE group by day 21 after treatment initiation. Scale bar = 1 cm. (**B**) Arthritis index scores over time. (**C**) Paw diameter (cm) over time as an indicator of edema. Data are presented as mean ± SD (n = 10 animals per group). Baseline measurements were obtained before CFA induction. Arthritis index and paw diameter were evaluated on days 7, 14, and 21 after treatment initiation. Statistical significance was determined using linear mixed-effects models with treatment group, time, and group × time interaction as fixed effects and animal identity as a random intercept to account for within-animal correlation. Baseline values were included as covariates. Post hoc pairwise comparisons were adjusted using the Bonferroni method (*p* < 0.05). NC, normal control; RA, arthritis control; Qu, quercetin; PSONE, pumpkin seed oil nanoemulsion; Qu-PSONE, quercetin-loaded pumpkin seed oil nanoemulsion.

To evaluate the therapeutic efficacy of the formulations, representative photographs of the rat paws are presented in Figure 2A, alongside with the clinical arthritis index and paw thickness at different time points are shown in Figure 2B,C. As expected, the CFA-induced adjuvant arthritis group showed a progressive increase in both parameters compared with the NC group throughout the study (*p* < 0.001 at all time points). Treatment with free Qu resulted in a moderate but significant reduction in arthritis index and paw thickness relative to the CFA-induced adjuvant arthritis group (*p* < 0.05 at day 7; *p* < 0.01 at day 21). Similarly, blank PSONE alone did not significantly improve clinical scores compared to RA at day 7, but showed a modest effect at day 21 (*p* < 0.05), suggesting a vehicle-related effect.

Remarkably, the Qu-PSONE group demonstrated the most robust therapeutic response. By day 21, the arthritis index was significantly reduced compared to the CFA-induced adjuvant arthritis group, and paw thickness returned to values not significantly different from those of the NC group. Moreover, Qu-PSONE showed significantly greater reductions in arthritis index and paw thickness compared with free Qu (*p* < 0.01) and blank PSONE (*p* < 0.05) at the late time point, indicating that the loaded nanoformulation may be associated with enhanced bioavailability and/or sustained release of Qu.

### 3.3. Radiographic Assessment and Quantitative Scoring of Ankle Joint Pathology in Experimental Groups

**Figure 3:**
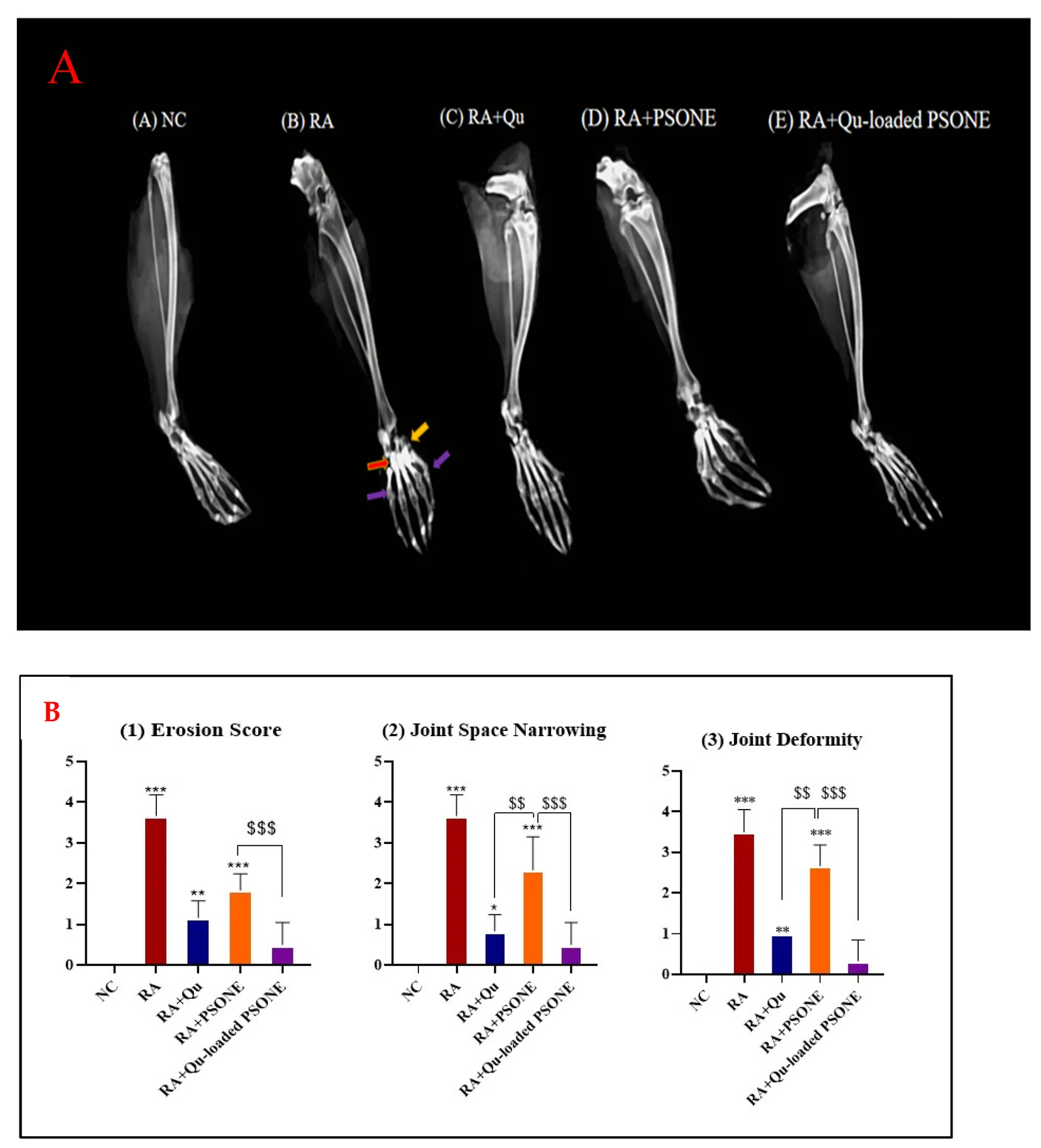
Radiographic assessment of ankle joint pathology. (**A**) Representative X-ray images of the hind limb/ankle joint from each experimental group: NC, RA, Qu, PSONE, and Qu-PSONE. Radiographs show characteristic RA-induced pathological changes, including extensive marginal erosions (yellow arrow), significant joint-space narrowing (red arrow), and visible bone deformity (purple arrow). Treatment with Qu, PSONE, and particularly Qu-PSONE showed progressive structural improvement and near-restoration of joint architecture. (**B**) Quantitative scoring of ankle joint pathology: (**B1**) bone erosion score, (**B2**) joint space narrowing score, and (**B3**) joint deformity score. Data are expressed as median (IQR) (n = 10 animals per group). Comparisons between groups were performed using the non-parametric Kruskal–Wallis test followed by Dunn’s multiple-comparison test, as radiographic scores represent ordinal data. * *p* < 0.05, ** *p* < 0.01, *** *p* < 0.001 versus the arthritis control group (RA); $$ *p* < 0.01, $$$ *p* < 0.001 for comparisons between treatment groups. NC, normal control; RA, arthritis control; Qu, quercetin; PSONE, pumpkin seed oil nanoemulsion; Qu-PSONE, quercetin-loaded pumpkin seed oil nanoemulsion.

Radiographic examination revealed marked structural alterations in the ankle joint of the RA group compared with controls Figure 3A. Control rats displayed normal bone morphology, intact cortical borders, and well-preserved joint space Figure 3A. In contrast, CFA-adjuvant arthritis induction produced severe pathological changes characterized by pronounced joint space narrowing, extensive subchondral bone erosion, and clear joint deformity Figure 3B (1,2,3). These abnormalities were consistently evident on the X-ray panel, particularly around the tibiotarsal joint, confirming successful establishment of CFA-induced adjuvant arthritis-associated damage.

Treatment with quercetin resulted in partial improvement of radiographic features, with visibly reduced erosion and moderately preserved joint spacing compared with the untreated CFA-induced adjuvant arthritis group. Pumpkin oil administration also demonstrated structural enhancement; however, residual narrowing and deformity remained apparent. Notably, the combined nano-formulated quercetin–pumpkin oil treatment produced the greatest radiographic recovery, with substantial restoration of joint alignment and attenuation of erosive lesions, approaching near-normal appearance Figure 3B (1,2,3).

Radiographic assessment of ankle joint pathology revealed significant structural damage in the RA group compared with the normal control group (NC) (*** *p* < 0.001 for all three parameters) (Figure 3). Treatment with Qu, PSONE, and Qu-PSONE significantly reduced erosion scores, joint space narrowing, and joint deformity compared with the RA group (* *p* < 0.05, ** *p* < 0.01, *** *p* < 0.001). Importantly, Qu-PSONE treatment resulted in significantly greater improvement compared with Qu alone ($$ *p* < 0.01 for joint space narrowing and joint deformity) and compared with PSONE alone ($$$ *p* < 0.001 for erosion score, joint space narrowing, and joint deformity).

### 3.4. Biochemical Results

CFA-induced adjuvant arthritis is an established acute/subacute animal model of joint inflammation that leads to joint deformity and systemic inflammation. This study tracks various signaling pathways and molecular mechanisms to identify a therapeutic intervention using CFA-induced adjuvant arthritis in a rat model. The overarching goal of this study is to examine and highlight the benefits of quercetin, pumpkin seed oil nanoemulsion, and quercetin-loaded pumpkin seed oil nanoemulsion in ameliorating CFA-induced adjuvant arthritis in rats.

#### 3.4.1. Effect of Qu, PSONE, and Qu-Loaded PSONE on Synovial JAK1, STAT3, and Nrf2 Protein Levels in CFA-Induced Adjuvant Arthritis

**Figure 4:**
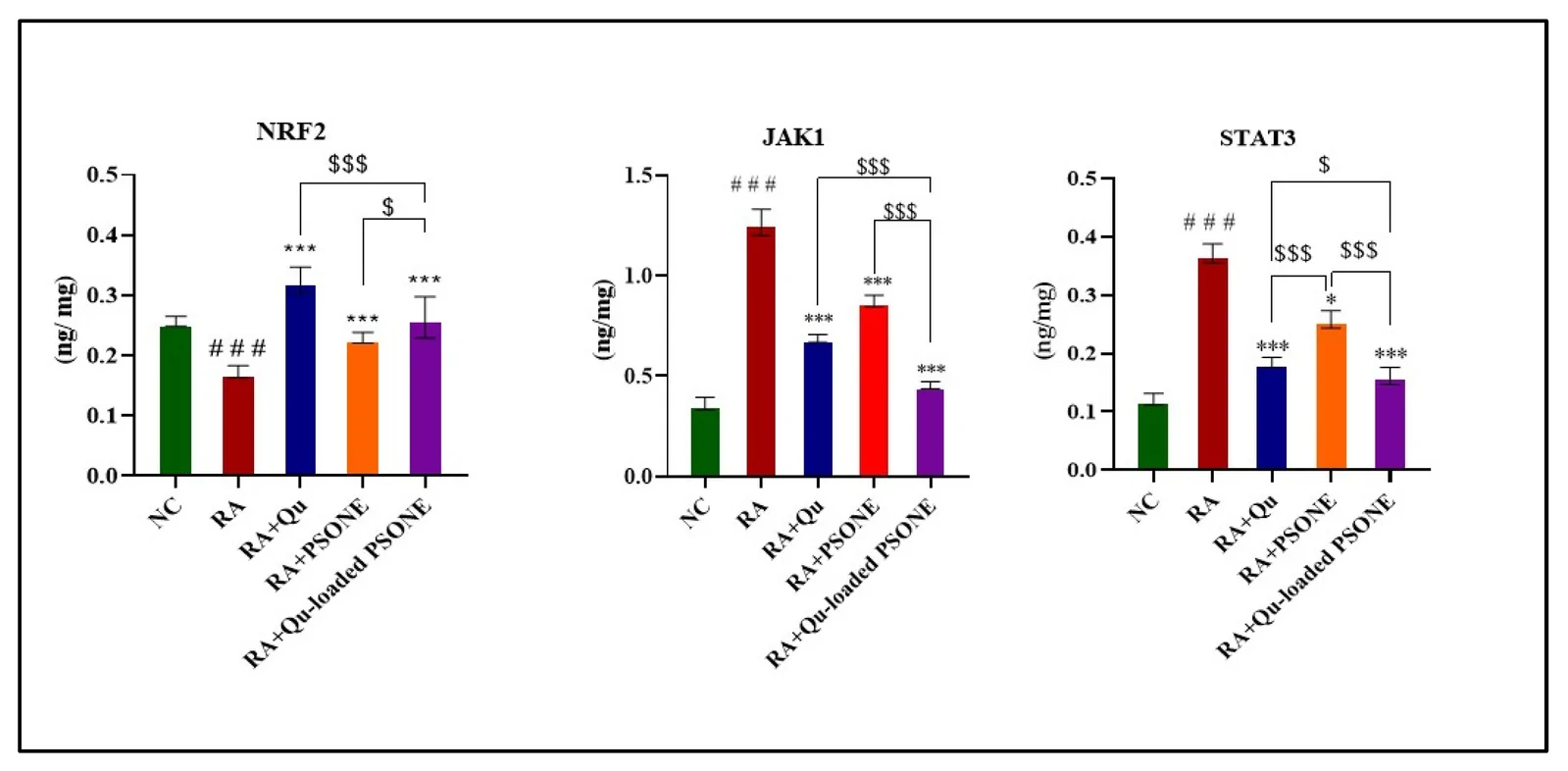
Effect of Qu, PSONE, and Qu-PSONE on Synovial Nrf2, JAK1, and STAT3 Protein Levels in CFA-Induced Adjuvant Arthritis, as determined by ELISA. JAK1: Janus Kinase 1; STAT3: Signal transducer and activator of transcription; NRF2: Nuclear factor erythroid 2-related factor 2. Data are presented as mean ± SD (n = 10 per group). Statistical significance was determined using one-way ANOVA followed by Tukey’s post hoc test for multiple comparisons. Significance is indicated on the graph as follows: ### *p* < 0.001 versus the normal control group (NC); * *p* < 0.05, *** *p* < 0.001 versus the CFA-induced adjuvant arthritis group (RA); $ *p* < 0.05, $$$ *p* < 0.001 for comparisons between treatment groups.

The present study evaluated Nrf2, JAK1, and STAT3 as key molecular targets regulating antioxidant defenses and inflammatory responses in joint pathology. As shown in Figure 4, the CFA-induced adjuvant arthritis control group exhibited a significant depletion of synovial Nrf2 protein levels alongside a marked elevation in total JAK1 and STAT3 synovial protein concentrations compared to the normal control group (### *p* < 0.001 for Nrf2 and JAK1; ## *p* < 0.01 for STAT3). Treatment with free Qu, blank PSONE, and Qu-loaded PSONE significantly reduced synovial JAK1 and STAT3 protein expression while restoring Nrf2 protein levels compared to the arthritis control group (*** *p* < 0.001 for Qu, PSONE, and Qu-PSONE across all three markers; * *p* < 0.05 for Qu-PSONE vs RA for STAT3). Notably, Qu-PSONE demonstrated the most pronounced therapeutic modulation compared to Qu and PSONE, with significantly greater restoration of Nrf2 and suppression of JAK1 and STAT3 than Qu alone (Figure 4).

#### 3.4.2. Comparative Anti-Inflammatory Effects of Qu, PSONE, and Qu-PSONE in CFA-Induced Adjuvant Arthritis

**Figure 5:**
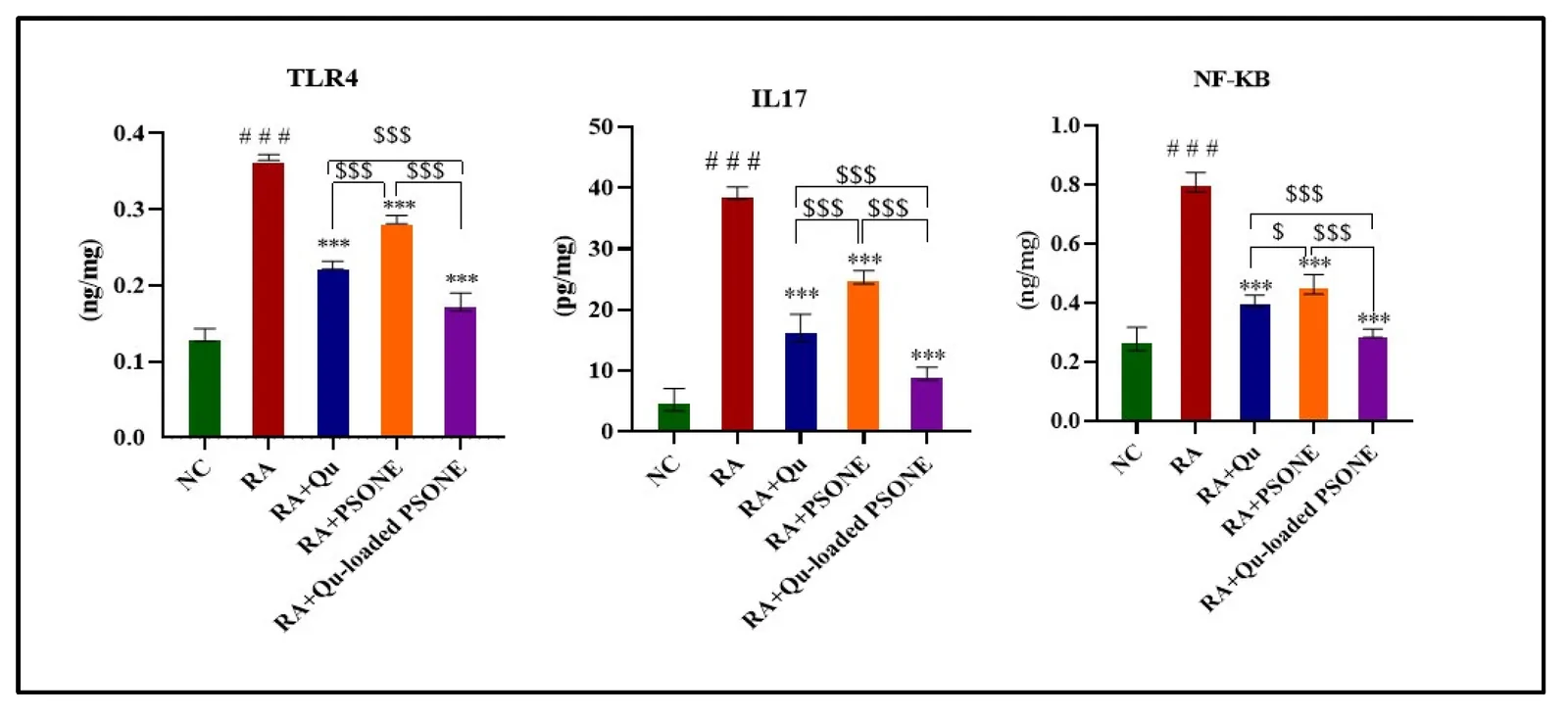
Effect of Qu, PSONE, and Qu-loaded PSONE on synovial inflammatory protein levels in CFA-induced adjuvant arthritis. TLR4: Toll-like receptor 4; IL-17: Interleukin-17; NF-κBp65: Nuclear factor kappa B, measured by ELISA. Data are presented as mean ± SD (n = 10 per group). Statistical significance was determined using one-way ANOVA followed by Tukey’s post hoc test for multiple comparisons. Significance is indicated on the graph as follows: ### *p* < 0.001 versus the normal control group (NC); *** *p* < 0.001 versus the CFA-induced adjuvant arthritis group (RA); $ *p* < 0.05, $$$ *p* < 0.001 for comparisons between treatment groups.

Figure 5 showed a significant elevation in synovial TLR4 levels in the RA group compared with the normal control group, with a significant decrease in all treated groups; the greatest decrease was observed in Qu-loaded PSONE compared with the other treated groups (Qu and PSONE). In addition, CFA-induced adjuvant arthritis induction resulted in a marked elevation of synovial IL-17 and NF-κBp65 concentrations. In contrast, oral administration of Qu, PSONE, and Qu-loaded PSONE reduced the elevated levels of NF-κBp65 and IL-17 compared with the untreated RA group. All evaluated proinflammatory markers were markedly elevated in arthritic rats (*p* < 0.001 versus NC), confirming successful induction of a robust inflammatory state. Each treated group showed significantly lower inflammatory cytokine levels than the untreated CFA-induced adjuvant arthritis group (*p* < 0.001 versus RA). Notably, Qu-loaded PSONE produced the greatest reduction in TLR4 and IL-17 compared with both Qu and PSONE (*p* < 0.001), while for NF-κBp65, Qu-loaded PSONE was significantly more effective than Qu (*p* < 0.05) and PSONE (*p* < 0.001).

#### 3.4.3. Qu, PSONE, and Qu-Loaded PSONE Modulate Osteoclastic Activation and Bone Erosion Biomarkers in RA-Induced Rats

**Figure 6:**
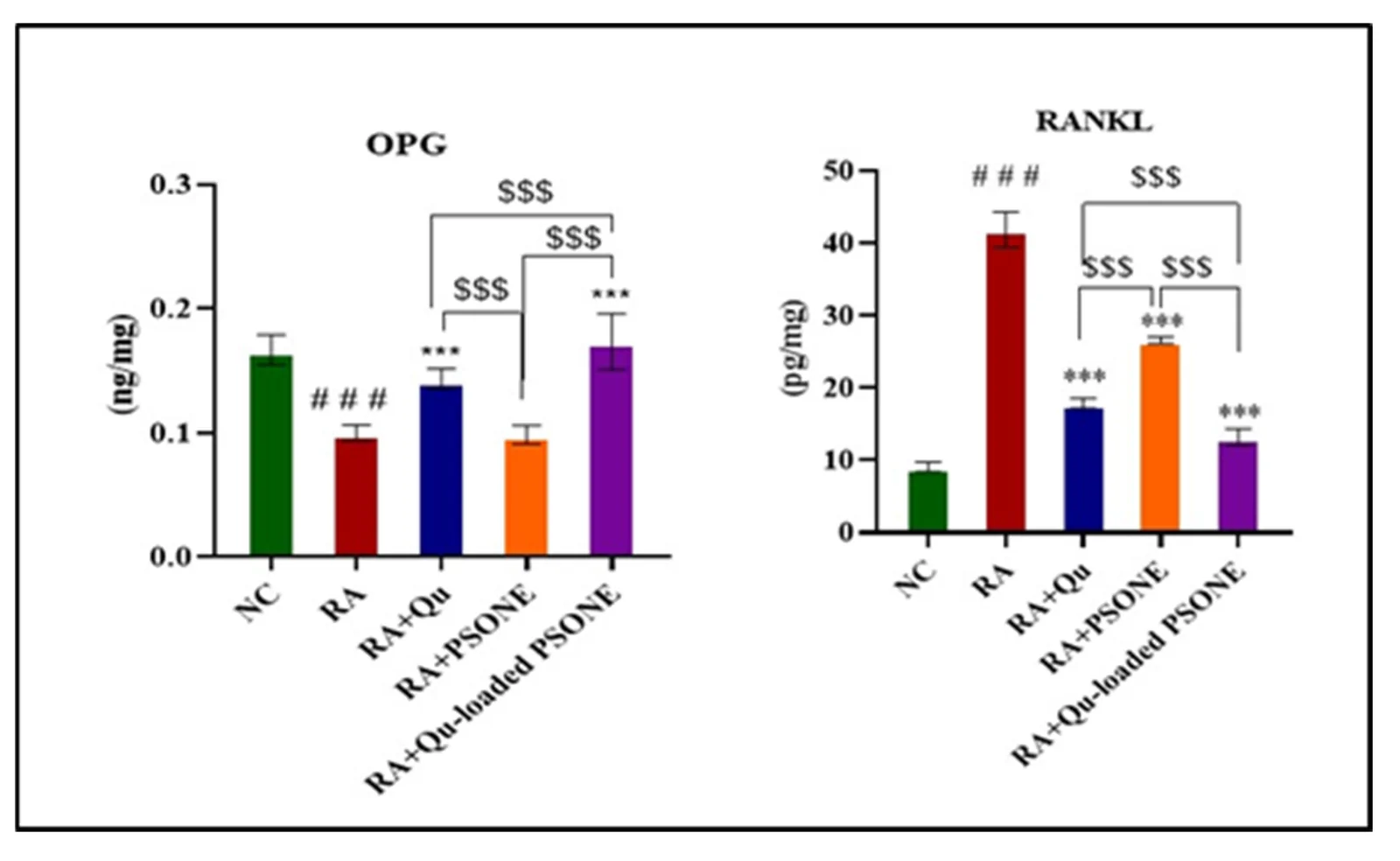
ELISA quantification of synovial RANKL and OPG levels in RA-induced rats following treatment with Qu, PSONE, and Qu-PSONE. RANKL: Receptor activator of nuclear factor κB ligand; OPG: Osteoprotegerin. Data are presented as mean ± SD (n = 10 per group). Statistical significance was determined using one-way ANOVA followed by Tukey’s post hoc test for multiple comparisons. Significance is indicated on the graph as follows: ### *p* < 0.001 versus the normal control group (NC); *** *p* < 0.001 versus the CFA-induced adjuvant arthritis group (RA); $$$ *p* < 0.001 for comparisons between treatment groups.

In the current study, we measured RANKL and OPG levels to investigate the anti-arthritic effects of Qu, PSONE, and Qu-loaded PSONE on osteoclastic activation and bone erosion associated with RA and induced by an inflammatory surge. Figure 6 shows that RA induction significantly increased synovial RANKL and significantly decreased synovial OPG levels, respectively, compared with the normal control group (### *p* < 0.001). Meanwhile, oral treatment with Qu, PSONE, and Qu-loaded PSONE significantly decreased RANKL levels and significantly increased OPG synovial levels compared with the untreated CFA-induced adjuvant arthritis control group (*** *p* < 0.001). Moreover, Qu-loaded PSONE treatment significantly restored OPG levels compared with both Qu and PSONE ($$$ *p* < 0.001). Treatment with Qu-loaded PSONE significantly attenuated the evoked inflammatory response and osteoclastic activation and thereby protected the joints compared with the normal control. The OPG and RANKL results are in line with radiographic results.

The CFA-induced adjuvant arthritis group displayed significant disturbances in both anabolic and catabolic bone-related factors (### *p* < 0.001), consistent with the destructive joint remodeling associated with inflammatory arthritis. Treatment effectively mitigated these alterations, leading to partial or complete normalization toward control levels. All treated groups differed significantly from the CFA-induced adjuvant arthritis (*** *p* < 0.001), with the Qu-PSONE group again demonstrating the most substantial correction of bone-remodeling abnormalities compared with Qu and PSONE ($$$ *p* < 0.001).

Taken together, one-way ANOVA followed by Tukey’s post hoc analysis indicated that the therapeutic interventions exerted anti-inflammatory and bone-protective effects. Among them, Qu-PSONE consistently produced the most favorable outcomes, indicating superior efficacy in modulating both inflammatory and structural components of arthritis.

#### 3.4.4. Influence of Qu, PSONE, and Qu-Loaded PSONE on GSDMD mRNA and miRNA-146a Expression in CFA-Induced Adjuvant Arthritis Rats

**Figure 7:**
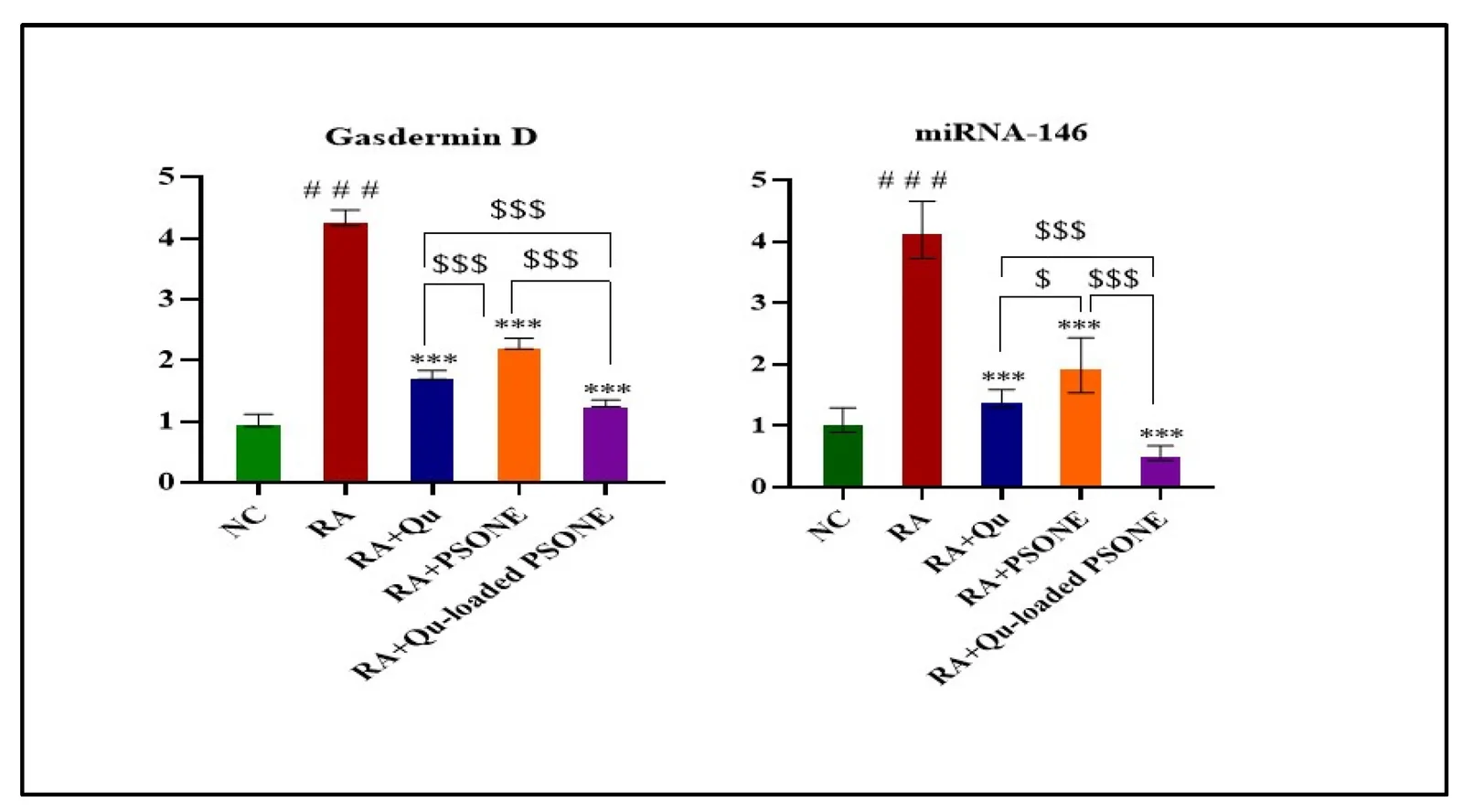
Effects of Qu, PSONE and Qu-PSONE on synovial GSDMD mRNA and miRNA-146a expression in CFA-induced arthritic rats, assessed by quantitative reverse-transcription PCR. Data are presented as mean ± SD; n = 10 animals per group. Statistical comparisons were performed using one-way ANOVA followed by Tukey’s multiple-comparison test. ### *p* < 0.001 versus the normal control group (NC); *** *p* < 0.001 versus the arthritis control group (RA); $ *p* < 0.05, $$$ *p* < 0.001 for comparisons between treatment groups.

The RA group showed significantly higher GSDMD and miRNA-146a transcript levels than the normal control group (### *p* < 0.001). Treatment with Qu, PSONE, and Qu-loaded PSONE significantly reduced the expression of both GSDMD and miRNA-146a compared with the RA group (*** *p* < 0.001). Notably, treatment with Qu-loaded PSONE significantly attenuated the gene expression of both GSDMD and miRNA-146a compared to treatment with either Qu or PSONE alone ($$$ *p* < 0.001 for GSDMD and miRNA-146a versus Qu and PSONE; $ *p* < 0.05 for miRNA-146a versus Qu), demonstrating a superior suppressive effect on these transcriptional targets, as shown in Figure 7.

### 3.5. Histopathological Evaluation of Joint Tissue (H&E) Results

**Figure 8:**
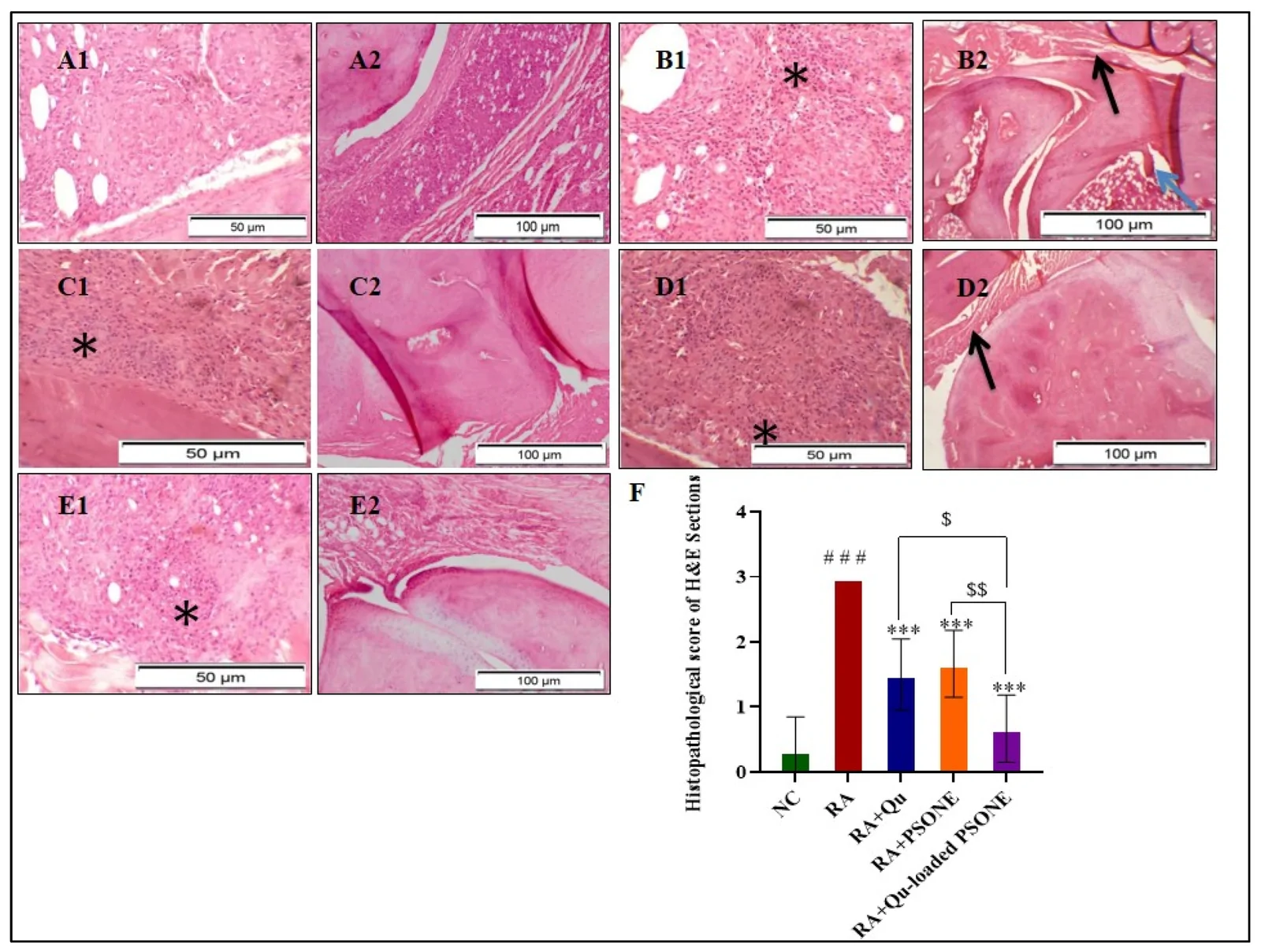
Histopathological assessment of left hind paw joint tissues following treatment with Qu, PSONE, and Qu-PSONE. Representative hematoxylin and eosin (H&E)-stained sections show (**A1**,**A2**) normal control (NC); (**B1**,**B2**) CFA-induced arthritis control (RA); (**C1**,**C2**) RA + Qu; (**D1**,**D2**) RA + PSONE; and (**E1**,**E2**) RA + Qu-PSONE. Black arrows indicate synovial hyperplasia, and blue arrows indicate bone erosion. Asterisks (*) within the photomicrographs indicate inflammatory cell infiltration in the subcutaneous tissue and do not denote statistical significance. (**F**) Histopathological scores, presented as medians with error bars indicating the interquartile range (25th–75th percentiles). Histological assessment was performed on 10 rats per group, with three randomly selected microscopic fields evaluated per rat. Statistical comparisons were performed using the Kruskal–Wallis test followed by Dunn’s multiple-comparison test. In panel F, ### *p* < 0.001 versus NC; *** *p* < 0.001 versus RA; $ *p* < 0.05 and $$ *p* < 0.01 indicate significant differences between the respective treatment groups.

The protective effects of Qu, PSONE, and Qu-loaded PSONE on joint architecture were assessed by histological analysis (Figure 8). Normal control rats exhibited intact synovial structure and regular articular cartilage arrangement (Figure 8A1,A2). In contrast, RA-model rats displayed hallmark features of rheumatoid arthritis, including severe inflammatory cell infiltration, synovial hyperplasia, cartilage degeneration, and bone erosion (Figure 8B1,B2). Notably, CFA-induced adjuvant arthritis-model rats treated with Qu (Figure 8C1,C2), PSONE (Figure 8D1,D2), and Qu-loaded PSONE (Figure 8E1,E2) demonstrated markedly reduced inflammation and preserved joint architecture compared to the untreated CFA-induced adjuvant arthritis group. Semi-quantitative histopathological scoring confirmed these observations, with the CFA-induced adjuvant arthritis group showing significantly higher scores than all other experimental groups (*p* < 0.05). Conversely, all treated groups showed significantly lower histopathological scores than the RA group (Figure 8F). Among the treatment groups, Qu-loaded PSONE showed the greatest reduction in histopathological score, indicating superior therapeutic efficacy.

### 3.6. Immunohistochemical Results

#### 3.6.1. TNF-α Immunohistochemical Staining

**Figure 9:**
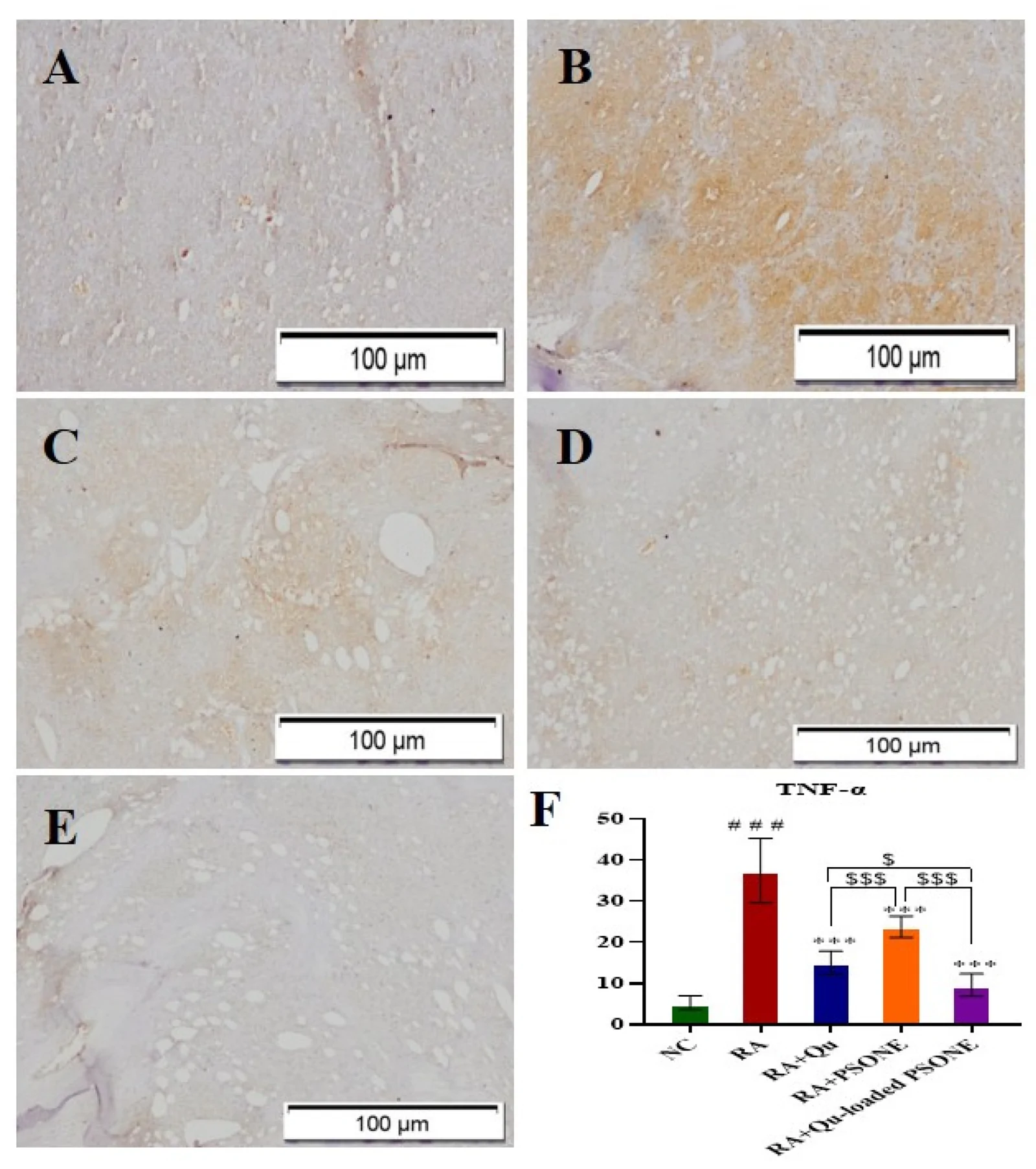
Immunohistochemical evaluation of TNF-α expression in ankle joint sections. Representative images show brown TNF-α-positive immunostaining in the following groups: (**A**) normal control (NC); (**B**) CFA-induced arthritis control (RA); (**C**) RA + Qu; (**D**) RA + PSONE; and (**E**) RA + Qu-PSONE. (**F**) Quantification of the percentage of TNF-α-positive area. Data are presented as medians with error bars indicating the interquartile range (25th–75th percentiles). Immunohistochemical quantification was performed on 10 rats per group, with three randomly selected microscopic fields evaluated per rat. Statistical comparisons were performed using the Kruskal–Wallis test followed by Dunn’s multiple-comparison test. In panel F, ### *p* < 0.001 versus NC; *** *p* < 0.001 versus RA; $ *p* < 0.05 and $$$ *p* < 0.001 indicate significant differences between the respective treatment groups. Scale bars: 100 μm.

TNF-α immunostaining was more pronounced in joint tissues from the CFA-induced arthritis control group than in normal controls (Figure 9B vs. Figure 9A). Treatment with Qu, PSONE, or Qu-PSONE reduced TNF-α immunoreactivity relative to the CFA-induced arthritis control group (Figure 9C–E). Morphometric analysis demonstrated significant differences in the percentage of TNF-α-positive area among the experimental groups. The arthritis control group exhibited the highest median percentage-positive area (### *p* < 0.001 vs. NC), whereas the Qu-PSONE group showed the lowest value among the treated groups (Figure 9F).

Comparisons between the treatment groups revealed that Qu-PSONE was significantly more effective than both Qu and PSONE alone. Specifically, the TNF-α-positive area in the Qu-PSONE group was significantly lower than in the Qu group ($ *p* < 0.05) and the PSONE group ($$$ *p* < 0.001). While Qu and PSONE both significantly reduced TNF-α expression compared to the arthritis control (*** *p* < 0.001 for both), their effects were less pronounced than that of the combined Qu-PSONE formulation.

#### 3.6.2. MMP-9 Immunohistochemical Staining

**Figure 10:**
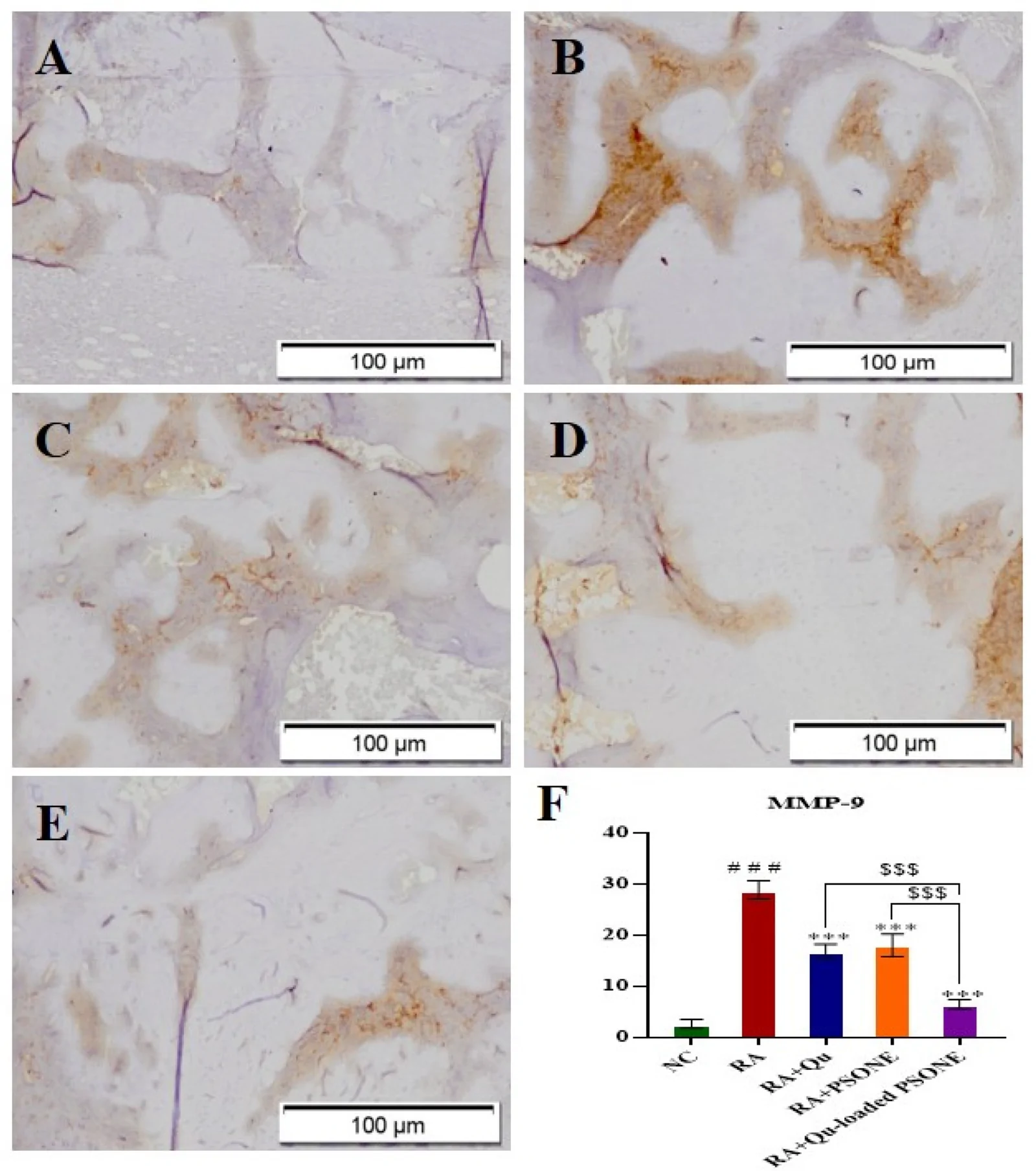
Immunohistochemical assessment of MMP-9 expression in ankle joint sections following treatment with Qu, PSONE, and Qu-PSONE. Representative photomicrographs show brown MMP-9-positive immunostaining in (**A**) normal control (NC); (**B**) CFA-induced arthritis control (RA); (**C**) RA + Qu; (**D**) RA + PSONE; and (**E**) RA + Qu-PSONE. (**F**) Quantification of the percentage of MMP-9-positive area. Data are presented as medians, with error bars indicating the interquartile range (25th–75th percentiles). Immunohistochemical quantification was performed on 10 rats per group, with three randomly selected microscopic fields evaluated per rat. Statistical comparisons were performed using the Kruskal–Wallis test followed by Dunn’s multiple-comparison test. In panel F, ### *p* < 0.001 versus NC; *** *p* < 0.001 versus RA; $$$ *p* < 0.001 indicates significant differences between the respective treatment groups. Scale bar: 100 μm.

MMP-9 immunostaining was markedly increased in the CFA-induced arthritis control group, with prominent perivascular and interstitial staining compared with the limited immunoreactivity observed in normal controls (Figure 10B vs. Figure 10A). Morphometric analysis demonstrated a significant overall difference in the percentage of MMP-9-positive area among groups (Kruskal–Wallis test, *p* < 0.001). The arthritis control group exhibited a significantly greater percentage-positive area than normal controls (### *p* < 0.001). Treatment with Qu, PSONE, or Qu-PSONE reduced MMP-9 immunoreactivity (Figure 10C–E). The Qu-PSONE group showed the lowest median percentage-positive area among the treated groups and did not differ significantly from normal controls.

Furthermore, Qu and PSONE also significantly reduced the percentage-positive area compared with the CFA-induced arthritis control group (*** *p* < 0.001 for both comparisons; Figure 10F). When comparing the treatments directly, Qu-PSONE demonstrated superior efficacy over PSONE alone, as indicated by a significant difference between these two groups ($$ *p* < 0.01). Although the Qu-PSONE group showed a lower median value than the Qu group, this specific pairwise comparison did not reach statistical significance in the post hoc analysis.

## 4. Discussion

RA is a progressive inflammatory autoimmune disease affecting approximately 1% of the population, with prevalence increasing with age. It is largely associated with elevated inflammatory reactions within synovial joints, affecting bone, ligaments, hyaline cartilage, tendons, and the synovial membrane, and it mostly affects the foot, hand, and wrist, although it may also affect larger joints [6]. CFA-induced adjuvant arthritis is an established acute/subacute animal model of joint inflammation that leads to joint deformity and systemic inflammation. This study tracks various signaling pathways and molecular mechanisms to identify a therapeutic intervention that improves quality of life in RA patients using CFA-induced adjuvant arthritis in a rat model. This investigation uses the Wistar rat CFA-induced adjuvant arthritis model because it resembles human RA in inflammatory cell infiltration, synovial membrane proliferation, and joint destruction [22]. Treating RA, a morbid inflammatory disorder, may involve a complex network of proinflammatory regulation of TNF-α, IL-6, NF-κB, and miRNA 146a [23].

The major goal of combining quercetin with PSONE was to increase quercetin’s biological effect against experimental inflammatory arthritis. The nanoemulsion for Qu delivery showed satisfactory thermal stability and bio accessibility [24]. Second-generation nanostructured lipid carriers are reported to enhance compound bioavailability and stability, while avoiding toxic organic solvents. Qu nanoparticles are more stable and effective than free Qu because they are released from the polymer matrix in a regulated manner. The increased biological activity observed after loading Qu in polymeric nanoparticles can be attributed to changes in membrane permeability, adsorption, and the formation of bonds between the nanoparticles and the cell membrane [25].

The anti-arthritic effect of the nanoscale size and lipid nature of the quercetin-loaded pumpkin seed oil nanoemulsion used in this study may be responsible for its enhanced therapeutic effect against CFA-induced adjuvant arthritis. In addition, providing a dual effect of a natural formulation and the use of a lipid nanocarrier rather than the use of toxic organic solvents.

Qu-PSONE dosages have been shown to reduce oxidative inflammation in CFA-induced adjuvant arthritis effectively. The results of this investigation suggest that Qu-PSONE may warrant further investigation as a potential adjunctive therapy for RA. However, clinical translation requires pharmacokinetic, toxicological, and clinical studies. Inflammation is a major pathological feature driving CFA-induced adjuvant arthritis, and inflammatory cytokines induce several abnormalities associated with joint-destructive mechanisms observed, such as bone erosion, membrane hyperplasia, and pannus formation [26]. CFA induction activates T lymphocytes, macrophages, and monocytes, resulting in the release of proinflammatory cytokines [27]. Several stress-sensitive and inflammatory pathways, including NF-κB, have been linked with RA. This pathway also regulates metabolism, the immune system, migration, survival, inflammation, and apoptosis, all of which are pathologically involved in CFA-induced adjuvant arthritis [28]. NF-κB regulates proinflammatory cytokines and chemokines, leading to persistent inflammation in RA [29]. NF-κB activation in fibroblasts and innate immune cells generates inflammatory cytokines and cells, perpetuating inflammation in the synovium. This study found that RA rats had higher levels of NF-κB and proinflammatory cytokines, indicating increased inflammation, which was confirmed by arthritis score [30].

In RA, oxidative stress depletes antioxidant defense and promotes the production of proinflammatory cytokines and mediators, including IL-17 and NF-κB, leading to synovitis. This oxido-inflammatory synergism increases the harmful effects of both factors, exacerbating RA progression and causing secondary problems [31]. In addition, RA can produce ROS through other enzymatic processes/sources, such as nitrite reductases, nitric oxide synthases, and phagocyte myeloperoxidase. Nrf2 plays an important role in cellular defense against oxidative damage, and its activation induces the expression of antioxidant genes and proteins that counter reactive radicals and oxidants [32]. As a result, reduced Nrf2 levels weaken the antioxidant machinery of cells and organs, leaving them vulnerable to oxidative attack. Nrf2 repression/knockout has been shown to enhance inflammation, cartilage degradation, oxidative load, and joint injury in RA models [30]. Herein, CFA-induced adjuvant arthritis rats revealed considerably lower Nrf2 levels, which were consistent with the previously mentioned study.

Oxidative stress and prooxidant radicals lead to synovial inflammation, hyperplasia and joint deformities. The level of Nrf2, the main regulator of the antioxidant pool, is reduced in the RA model. Furthermore, Nrf2 inactivation and antioxidant depletion further activate prooxidant and proinflammatory signaling through NF-κB pathway activation, leading to chronic inflammation and worsening joint damage in RA [33]. Thus, fostering antioxidant pool and activating the Nrf2 is cardinal in experimental inflammatory arthritis condition to derail the ramifications of RA. The combined effect of quercetin and pumpkin seed oil nanoemulsion in boosting the antioxidant pool and hurdling oxidative stress and inflammatory response activation can be elucidated by previous studies that elucidate the antioxidant and anti-inflammatory effect of quercetin caused by activating a Nrf2/HO-1, SOD, and GPX and inhibiting NF-κB, TNF-α, IL-6, and IL-1β [34]. Additionally, a recent study reported that the administration of pumpkin seed oil nurtures antioxidant defenses and reduces inflammatory signaling due to its content of antioxidants, carotenoids, tocopherols, omega-3, zinc, selenium, and phytosterols [35], which were found to activate Nrf2, SOD, GPx, and GSH and reduce levels of TNF-α, IL-1β, and MDA. These results explain the antioxidant and anti-inflammatory effects of both quercetin and pumpkin seed oil [36].

Confirming these results, Yazdi M. et al. [9] examined the antioxidant and anti-inflammatory effects of quercetin and quercetin-loaded pumpkin seed oil nanoemulsion on testosterone-induced prostatic hyperplasia in rats. Results showed that the quercetin-loaded pumpkin seed oil nanoemulsion administration was effective in restoring the antioxidant pool and inhibiting inflammatory response by increasing SOD, GPX, and TAC while decreasing lipid peroxidation and TNF-α levels.

Oxidative stress activates several signaling pathways involved in structural changes of joints by affecting chondrocytes and synovial cells’ survival [33]. ROS generation causes oxidative stress and the activation of pyroptosis, a type of cell death triggered by ROS activation of the inflammatory cascade. Activating NF-κB p65 stimulates pyroptosis [37]. In the present study, the measured pyroptosis gene was Gasdermin D, which was upregulated in CFA-induced adjuvant arthritis and downregulated by administration of quercetin and PSONE. Herein, quercetin exerts an anti-inflammatory effect by suppressing *GSDMD* mRNA expression, thereby dampening the transcriptional priming of the pyroptotic pathway. While functional execution of pyroptosis requires proteolytic cleavage of GSDMD, this transcriptional downregulation suggests a potential mechanism by which quercetin limits the signaling capacity of this inflammatory cell death pathway.

Quercetin is a targeted treatment for RA because it affects inflammatory cells and molecules by inhibiting inflammatory processes. Quercetin boosted antioxidant enzyme protection in joint tissue by reducing free radical generation and consequently raising Nrf2 levels, as reported by [38].

Remarkably, cold-pressed pumpkin seed oil is known for its broad therapeutic benefits, including antioxidant, anti-inflammatory, antiviral, immunomodulatory, anticancer, hepatoprotective, and antidiabetic properties, which are traditionally attributed to its rich content of unsaturated fatty acids—primarily linoleic and oleic acids—as well as bioactive phytochemicals [39]. Notably, bioactive components of pumpkin seed oil described in the literature include tocopherols, carotenoids, and phytosterols, such as β-sitosterols and other sterols [40]. In addition, omega-3, which was previously reported for its putative mechanism in inhibiting inflammatory signaling activation in the RA model, inhibits TLR-4, TH17, NF-κB, and osteoclast activity [41]. These findings interpret the results of the present study.

Β-sitosterol, one of the main active components of pumpkin seed oil, was reported for its suppressive effect on bone resorption via targeting the OPG/RANKL pathway. Recent studies examined and explained the Β-sitosterol effect on oxidative stress and cartilage repair in the osteoarthritis model [42].

Previous studies performed chromatographic and GC-MS characterizations of pumpkin seed oil demonstrate a rich lipophilic matrix dominated by essential unsaturated fatty acids—primarily linoleic (42.5–52.0%) and oleic (28–38%) acids—alongside high concentrations of total tocopherols (gamma and alpha tocopherol), phytosterols (beta-sitosterol), and phenolic compounds. Reporting that these constituents exhibit potent radical-scavenging and anti-inflammatory properties [43]. This provides a robust pharmacological rationale for using pumpkin seed oil as the lipid carrier in nanoemulsified delivery systems.

Although we did not quantitatively profile individual bioactive components in the batch used in this study, these literature-documented constituents—potentially acting alongside enhanced intestinal absorption mediated by the self-emulsifying drug delivery system—provide a plausible hypothesis for the observed therapeutic benefits. Future analytical characterization (e.g., GC-MS or HPLC quantification) will be required to confirm the precise bioactive drivers.

Quercetin promotes bone remodeling through bidirectional crosstalk between the OPG/RANKL and NRF2/NF-κB axes. Thus, it inhibits oxidative activation of the inflammatory response, suppresses inflammatory cytokines acting on RANKL/OPG, and inhibits bone loss [44].

In the current study, PSONE administration to arthritic rats significantly improved inflammatory and oxidative stress indicators and reduced synovial inflammation. These effects likely stem from PSONE’s antioxidant and anti-inflammatory ingredients, including tocopherols, carotenoids, phytosterols, and phenolic compounds [45]. These compounds could provide the oil with antioxidant and anti-inflammatory properties. PSONE, including α, δ, and γ-tocopherol, may significantly improve alterations in RA rats. PSO’s antioxidant and anti-inflammatory properties may be attributed to its high phenolic content and tocopherol and β-carotene concentrations. Antioxidants are crucial in treating arthritis in rats [46].

The RANKL/OPG pathway was found to be involved in RA pathogenesis. Macrophages trigger the osteoclast differentiation factor RANKL, which fuses with RANK on osteoclasts [47]. Osteoblasts produce OPG, which blocks the RANK receptor and limits RANKL activity. Previous studies have shown that high ROS levels play an important role in RANKL-induced osteoclastogenesis [48] by stimulating RANKL expression. Studies have shown that RA incidence is associated with a substantial rise in RANKL expression through NF-κB activation [49].

Osteoclasts bind to the matrix, releasing hydrochloric acid and cathepsin K (a proteolytic enzyme), resulting in severe joint deterioration, pannus development, inflammatory exudate in the joint space, and focal cartilage necrosis. The current findings demonstrated that CFA dramatically reduced tissue OPG levels while significantly increasing tissue RANKL levels. As a result, the RANKL/OPG ratio increased in CFA-induced arthritis as considered the main factor of osteoclast differentiation and functional activity [14]. Oral administration of Qu, PSONE, and Qu-loaded PSONE to rats with RA significantly decreased RANKL while significantly increasing OPG levels, resulting in a reduction in the RANKL/OPG ratio, indicating decreased osteoclast activity. It also increased osteoblast activity and improved joint damage, with reduced pannus formation [50]. Qu-PSONE reduced the RANKL/OPG ratio to levels that did not differ significantly from normal controls. The excipient effect of quercetin and pumpkin seed oil nanoemulsion may enhance its antioxidant and anti-inflammatory effects; therefore, this formula may provide a strong promise for managing and controlling CFA-induced adjuvant arthritis development.

RA is a systemic autoimmune illness caused by increased activation and production of proinflammatory cytokines, leading to Th17 infiltration and further IL-17 production in synovial tissues. Inflammatory cytokines activate osteoclasts through receptor activator of nuclear factor κB ligand (RANKL). This binding also triggers the release of numerous inflammatory mediators by activating inflammatory genes and causing tissue damage [51].

The JAK/STAT signaling pathway plays an integral role in immune integrity and inflammation. RA-associated synovitis and bone deformities are reported to be related to JAK/STAT activation, which is in concordance with the results of the present study. Studies have reported that elevated JAK and STAT levels are the main drivers of synovial and systemic inflammation. The JAK/STAT pathway is a multifunctional machinery that regulates transcription of various genes involved in apoptosis, inflammation, and immunity. Here, inhibiting the JAK/STAT pathway represses major proinflammatory cytokines, such as IL-17 [52].

Noncoding RNAs, such as miRNAs, have gained particular interest in this era because they are involved in the occurrence and progression of several diseases. Several miRNAs regulate the crosstalk between inflammation and bone integrity [14]. Previous research shows that miR-146a is highly upregulated in rheumatoid arthritis as part of an inflammation-inducible feedback loop. Specifically, activation of the NF-kappaB pathway drives the transcription of miR-146a in an attempt to limit inflammation. Consequently, elevated miR-146a levels in untreated arthritis serve as a molecular footprint of severe, ongoing inflammatory stress rather than a primary pathogenic driver [23]. This illustration confirmed the results of the present study.

The results of the present study demonstrate that the severe inflammatory response observed in the CFA-induced adjuvant arthritis model is driven by NF-κBp65 activation and a concomitant surge in proinflammatory cytokines, which subsequently induces a compensatory upregulation of miRNA-146a. Consequently, the reduction in miR-146a following Qu-loaded PSONE treatment reflects the successful suppression of the primary upstream inflammatory burden (NF-kappa B p65 activation), which diminishes the cellular demand for this compensatory stress response.

In the present study, untreated arthritic rats exhibited elevated miRNA-146a expression, which represents an inducible, NF-κB-p65-driven compensatory response to brake systemic inflammation rather than a driver of pathogenesis. Consequently, the significant reduction in miRNA-146a following Qu-loaded PSONE treatment reflects a diminished upstream inflammatory burden and suppressed NF-kappa B p65 activation, thereby lowering the requirement for compensatory miRNA-146a induction. While these correlative data highlight miRNA-146a as a sensitive biomarker of therapeutic response in RA, future studies addressing functional gain- or loss-of-function using mimics or antagomirs are necessary to establish whether miRNA-146a plays a direct causal role in mediating the anti-inflammatory efficacy of Qu-loaded PSONE.

Quercetin, reported in several studies for its immunomodulatory, antioxidant, and anti-inflammatory effects, treats several comorbidities through a multitarget approach by influencing the JAK/STAT pathway, inhibiting NF-κB, TNF-α, and TLR-4, and activating the Nrf2/HO-1 pathway [53].

Thus, this study highlights the potential of combining quercetin with a pumpkin seed oil nanoemulsion for modulating inflammatory and oxidative stress pathways in experimental arthritis. This formulation effectively attenuates the inflammatory cascade by suppressing synovial NF-κBp65, TLR-4, and IL-17 signaling. Consequently, the treatment-induced resolution of this inflammatory surge lowers the requirement for compensatory miRNA-146a expression, thereby resetting homeostatic signaling balance and halting the propagation of arthritic tissue damage.

The histopathological and immunohistochemical findings provided complementary evidence supporting the therapeutic effects observed in this study. H&E examination confirmed substantial joint pathology in the RA group, characterized by inflammatory cellular infiltration, synovial alterations, and structural tissue damage, whereas treatment with Qu, PSONE, and particularly Qu-loaded PSONE markedly ameliorated these changes. This is consistent with previous studies demonstrating that quercetin reduces arthritis-associated pathological alterations, including a recent report in collagen-induced arthritic rats and a systematic review confirming significant reductions in histopathological scores in experimental RA [54].

The immunohistochemical assessment of TNF-α and MMP-9 provided molecular support for these morphological observations. The pronounced TNF-α immunoreactivity in untreated arthritic joints aligns with its established role as a major mediator of synovial inflammation; notably, reduced TNF-α expression is consistent with reported quercetin-associated anti-rheumatic effects [55]. Similarly, increased MMP-9 immunoreactivity is compatible with enhanced extracellular matrix remodeling, and previous work has shown that quercetin suppresses MMP-9 and other matrix-degrading mediators. Thus, the simultaneous reduction in TNF-α and MMP-9 following treatment, particularly in the Qu-loaded PSONE group, suggests that histological improvement was accompanied by attenuation of both inflammatory activation and matrix-remodeling processes.

Notably, Qu-loaded PSONE produced the most pronounced improvement in both H&E morphology and immunohistochemical expression of TNF-α and MMP-9, with immunoreactivity approaching that of the healthy control group. This agrees with previous studies showing that quercetin nanoformulations provide enhanced anti-arthritic activity compared with free quercetin, including quercetin-loaded chitosan nanoparticles and quercetin-loaded stearic acid nanoparticles [56], both of which improved arthritic histopathology and reduced TNF-α and other inflammatory mediators in arthritic rats.

While the nanoformulation exhibited superior biological activity compared to individual components, pharmacokinetic, plasma concentration-time, biodistribution, and in vitro dissolution profiles were not determined in the present study. Consequently, potential contributions from enhanced bioavailability, altered tissue distribution, or sustained drug release remain hypotheses that require explicit pharmacokinetic validation in future studies.

Taken together, the concordant H&E and immunohistochemical findings strengthen the interpretation that Qu-loaded PSONE exerts a favorable joint-protective effect in experimental inflammatory arthritis. However, these immunohistochemical data demonstrate changes in protein immunoreactivity and should be interpreted as supportive evidence rather than definitive proof of molecular causality. Overall, these findings align with recent experimental evidence supporting quercetin as a promising anti-arthritic agent and further suggest that incorporating it into PSONE may enhance its therapeutic performance.

### Study Limitations

While this study demonstrates promising therapeutic efficacy, several limitations should be acknowledged. The absence of a standard clinical reference drug precludes direct benchmarking of the relative efficacy of the tested formulations. Furthermore, comprehensive pharmacokinetic and analytical profiling including plasma concentration, time profiles, biodistribution, direct bioavailability, and batch chemical characterization was not performed. Consequently, improvements in bioavailability and the precise contribution of individual constituents are inferred from downstream pharmacodynamic and histopathological outcomes rather than direct pharmacokinetic validation. Future studies incorporating standard clinical comparators and rigorous analytical assessments are warranted to fully validate the translational potential of Qu-PSONE.

## 5. Conclusions

Overall, these findings demonstrate the promising anti-arthritic potential of quercetin, particularly when delivered via pumpkin seed oil nanoformulation. The Qu-loaded PSONE formulation favorably modulated inflammatory and molecular biomarkers, including miRNA-146a and Gasdermin D, suggesting enhanced regulation of the inflammatory response and protection against CFA-induced adjuvant arthritis-associated joint damage. These findings support Qu-loaded PSONE as a promising multifunctional nanotherapeutic approach for CFA-induced adjuvant arthritis. Further computational and molecular studies are warranted to validate its potential targets and underlying mechanisms.

## Figures and Tables

**Table 1:** Primer Sequences.

Gene	Primer Sequence	
GSDMD	Forward Sequence	5′ AGATCGTGGATCATGCCGTC 3′
	Reverse Sequence	5′ CCCGATGGAATGGAGTACGG 3′
MiRNA-146a	Forward Sequence	5′ TGAGAACTGAATTCCATGGGTT 3′
	Reverse Sequence	Universal Reverse Primer (Kit Provided)
U6 snRNA	Forward Sequence	5’CTCGCTTCGGCAGCACA3’
	Reverse Sequence	5’ AACGCTTCACGAATTTGCGT 3’
GAPDH	Forward Sequence	5′ CGGAGTCAACGGATTTGGTCGTAT 3′
	Reverse Sequence	5′ AGCCTTCTCCATGGTGGTGAAGAC 3′

**Table 2:** Results of characterization of Nanoformulations.

Sample	Mean Particle Size (nm)	Zeta Potential (mv)	PDI
Quercetin-loaded nanoemulsion pumpkin seed oil	101.6 ± 67.5	−35	0.422 ± 0.05
Nanoemulsion pumpkin seed oil without quercetin	88 ± 54.8	−23.4	0.240 ± 0.022

## Data Availability

The original contributions presented in this study are included in the article. Further inquiries can be directed to the corresponding authors.

## Abbreviations

The following abbreviations are used in this manuscript:

**Abbreviation**	**Full Name**
RA	Rheumatoid arthritis
Qu	Quercetin
PSONE	Pumpkin seed oil nanoemulsion
Qu-loaded PSONE	Quercetin-loaded pumpkin seed oil nanoemulsion
TNF-α	Tumor necrosis factor-alpha
MMP-9	Matrix metalloproteinase-9
JAK1	Janus kinase 1
STAT3	Signal transducer and activator of transcription 3
Nrf2	Nuclear factor erythroid 2-related factor 2
NF-κBp65	Nuclear factor kappa B p65
IL-17	Interleukin-17
miRNA-146a	MicroRNA-146a
H&E	Hematoxylin and eosin
GSDMD	Gasdermin D
